# Deciphering cyanobacterial phenotypes for fast photoautotrophic growth via isotopically nonstationary metabolic flux analysis

**DOI:** 10.1186/s13068-017-0958-y

**Published:** 2017-11-16

**Authors:** Mary H. Abernathy, Jingjie Yu, Fangfang Ma, Michelle Liberton, Justin Ungerer, Whitney D. Hollinshead, Saratram Gopalakrishnan, Lian He, Costas D. Maranas, Himadri B. Pakrasi, Doug K. Allen, Yinjie J. Tang

**Affiliations:** 1 Department of Energy, Environmental and Chemical Engineering, Washington University, St. Louis, MO 63130 USA; 2 Department of Biology, Temple University, Philadelphia, PA 19122 USA; 3 Donald Danforth Plant Science Center, St. Louis, MO 63132 USA; 4 Department of Biology, Washington University, St. Louis, MO 63130 USA; 5 Department of Chemical Engineering, The Pennsylvania State University, University Park, PA 16802 USA; 6 United States Department of Agriculture, Agricultural Research Service, St. Louis, MO 63132 USA

**Keywords:** ^13^C labeling experiments, Channeling, Glycogen, Metabolites, Photobioreactor, Energy charge

## Abstract

**Background:**

*Synechococcus elongatus* UTEX 2973 is the fastest growing cyanobacterium characterized to date. Its genome was found to be 99.8% identical to *S. elongatus* 7942 yet it grows twice as fast. Current genome-to-phenome mapping is still poorly performed for non-model organisms. Even for species with identical genomes, cell phenotypes can be strikingly different. To understand *Synechococcus* 2973’s fast-growth phenotype and its metabolic features advantageous to photo-biorefineries, ^13^C isotopically nonstationary metabolic flux analysis (INST-MFA), biomass compositional analysis, gene knockouts, and metabolite profiling were performed on both strains under various growth conditions.

**Results:**

The *Synechococcus* 2973 flux maps show substantial carbon flow through the Calvin cycle, glycolysis, photorespiration and pyruvate kinase, but minimal flux through the malic enzyme and oxidative pentose phosphate pathways under high light/CO_2_ conditions. During fast growth, its pool sizes of key metabolites in central pathways were lower than suboptimal growth. *Synechococcus* 2973 demonstrated similar flux ratios to *Synechococcus* 7942 (under fast growth conditions), but exhibited greater carbon assimilation, higher NADPH concentrations, higher energy charge (relative ATP ratio over ADP and AMP), less accumulation of glycogen, and potentially metabolite channeling. Furthermore, *Synechococcus* 2973 has very limited flux through the TCA pathway with small pool sizes of acetyl-CoA/TCA intermediates under all growth conditions.

**Conclusions:**

This study employed flux analysis to investigate phenotypic heterogeneity among two cyanobacterial strains with near-identical genome background. The flux/metabolite profiling, biomass composition analysis, and genetic modification results elucidate a highly effective metabolic topology for CO_2_ assimilatory and biosynthesis in *Synechococcus* 2973. Comparisons across multiple *Synechococcus* strains indicate faster metabolism is also driven by proportional increases in both photosynthesis and key central pathway fluxes. Moreover, the flux distribution in *Synechococcus* 2973 supports the use of its strong sugar phosphate pathways for optimal bio-productions. The integrated methodologies in this study can be applied for characterizing non-model microbial metabolism.

**Electronic supplementary material:**

The online version of this article (10.1186/s13068-017-0958-y) contains supplementary material, which is available to authorized users.

## Background

Efforts toward a sustainable bio-based economy have focused on phototrophic hosts for the production of chemicals from CO_2_ and light. Cyanobacteria are of special biotechnological interest due to their metabolic flexibility, unicellular anatomy, and high photosynthetic efficiency. Additionally, cyanobacteria can use flue gas from power plants to mitigate CO_2_ emissions that contribute to climate change [1]. To achieve metrics required for commercialization, cyanobacterial photo-biorefineries must have comparable biosynthesis capability to commonly used heterotrophic organisms. Recently, *Synechococcus elongatus* UTEX 2973 was isolated, whose growth rate reaches a doubling time of 2 h under high light and high CO_2_ conditions [2]. In comparison, under optimal growth conditions, *S. elongatus* PCC 7942 exhibits a doubling time of ~ 5 h although its genome sequence is 99.8% identical to *Synechococcus* 2973 (55 single nucleotide polymorphisms and a large 188 kb inversion between *Synechococcus* 2973 and 7942) [2]. To understand how cyanobacteria achieve maximal growth rates, this study describes *Synechococcus* 2973 flux topology under both optimal and suboptimal growth conditions.

Metabolic flux analysis (MFA) can provide a quantitative description of the metabolic network, link genome profiling to phenome analysis, and reveal pathway regulations through comparative studies. Currently, the cyanobacterial strain, *Synechocystis* sp. PCC 6803 (doubling time ~ 8 h), is considered to be the model cyanobacterium whose metabolism has been extensively profiled by flux analysis tools [3–6]. *Synechocystis* 6803 has significant flux through malic enzyme and oxidative pentose phosphate pathways (OPPP) under the photoautotrophic and photomixtrophic conditions. It also operates a cyclic TCA cycle via the γ-aminobutyric acid (GABA) shunt, which forms succinate through the intermediates glutamate and succinate semialdehyde, despite the missing enzyme from 2-oxoglutarate to succinyl-CoA [7]. To profile *Synechocystis* 6803 photoautotrophic metabolism, ^13^C-bicarbonate pulse experiments and isotopically nonstationary metabolic flux analysis (INST-MFA) were developed [3]. Using the software package, INCA, mass isotopomer data from dynamic labeling experiments can be used to quantify fluxes without the need to precisely determine metabolite pool sizes (which are fitted as parameters to account for transient labeling data) [8], and is therefore more convenient than other flux profiling methods [9]. In the current study, INST-MFA, gene knockouts, and metabolite analysis were performed to obtain insights into the physiology and metabolic regulations of *Synechococcus* 2973 under different bioreactor conditions. Meanwhile, aspects of biomass composition were measured to reveal changes in macromolecule tradeoffs that correlate to cell growth and bioreactor conditions [10]. The outcome highlights the advantages and hurdles of establishing *Synechococcus* 2973 as a new platform organism for bioproduction. The comparative studies among metabolisms of different cyanobacterial strains may also offer new insights into flux dependency on adaptive evolution.

## Results

### *Synechococcus* 2973 growth and biomass compositions

**Fig. 1:**
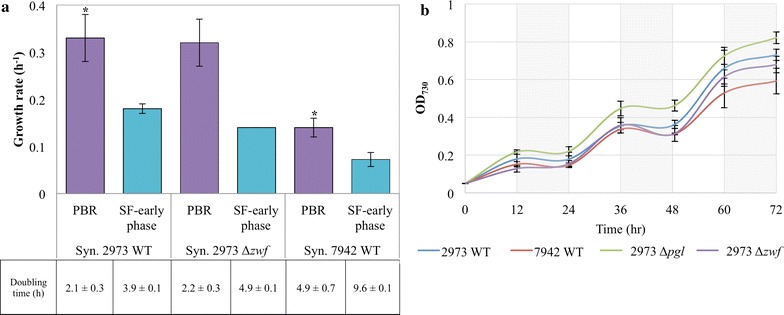
Growth performances of cyanobacterial species. **a** Growth rate of *Synechococcus* 2973, *Synechococcus* 7942 and the *Synechococcus 2973* Δ*zwf* mutant in PBR and SF conditions under continuous light conditions. Doubling times in hours are reported below for each strain. ^†^ *Synechococcus* 7942 was grown at 300 μmol photons/m^2^s. Standard deviations are a result of 3–5 biological replicates. *p value < 0.02 between *Syn.* 2973 and 7942 PBR conditions using two-tailed equal variance Student’s t test. **b** Diurnal growth curve and rates of *Synechococcus* strains under SF conditions. 12-h diurnal growth curve of *Synechococcus* 2973 WT*, Synechococcus* 7942 WT*, Synechococcus* 2973 *∆zwf* and *Synechococcus* 2973 *∆pgl.* Standard deviations are based on three biological replicates

In optimal photobioreactor (PBR, 500 μmol photons/m^2^s continuous light) conditions [2], *Synechococcus* 2973 exhibits very rapid growth (0.33 ± 0.05 h^−1^; Fig. 1a). For comparison, maximal growth rate of *Synechococcus* 7942 was 0.14 ± 0.02 h^−1^ at 300 μmol photons/m^2^s continuous light due to photo-inhibition at greater light intensities. *Synechococcus* strains were also grown under suboptimal light conditions in shake flasks (100 μmol photons/m^2^s continuous light or diurnal light irradiation; Fig. 1b). Suboptimal cultivations greatly impaired cyanobacterial photosynthesis, while *Synechococcus* 2973 still demonstrated moderately faster growth rate than *Synechococcus* 7942. Based on growth and biomass composition analyses, *Synechococcus* 2973 assimilated ~ 12.2 mmol-C/gDCW/h in the PBR but only ~ 6.7 mmol-C/gDCW/h under the shaking flask (SF) conditions. The estimated values of carbon fixation were further confirmed by the measured net CO_2_ uptake rate using gas chromatography methods (Additional file 1: Figure S1) [11]. This study additionally considers two *Synechococcus* 2973 mutants (∆*zwf* and ∆*pgl*) whose genes from the oxidative pentose phosphate pathway (OPPP) were removed. The two mutants showed unimpaired growth rates compared to the wild-type strain in our tested conditions (Fig. 1).

The composition of *Synechococcus* 2973 biomass was measured in both PBR and SF conditions under continuous light (Additional file 1: Part 1). The total protein was ~ 50% of cyanobacterial biomass, while protein from the PBR had more glutamate/glutamine than that from the SF (*p* value 0.054 using two-tailed equal variance Student’s t Test). *Synechococcus* 2973 contained 9 ± 1% lipids and 1.5 ± 0.5% glycogen in the PBR and 13 ± 2% lipid and 6.0 ± 1.0% glycogen in SF conditions. For comparison, *Synechococcus* 7942 biomass composition was quantified under optimal PBR conditions and comprised 11 ± 1% lipids, 13 ± 4% glycogen, and 41 ± 0.4% protein. Proteinogenic glutamate/glutamine content in *Synechococcus* 7942 was significantly lower (p  < 0.05) than that in *Synechococcus* 2973 biomass regardless of conditions. Fatty acid compositions of lipids in both strains were predominantly C16:1 and C16:0 fatty acids (85%), followed by C18:1 and C18:0. Relevant to photosynthetic light-harvesting, less chlorophyll *a* was produced in PBR conditions (4.4 ± 0.5 µg/mL/OD_730_ for 2973; 5.2 ± 0.6 µg/mL/OD_730_ for 7942) compared to SF (7.4 ± 0.3 µg/mL/OD_730_ for 2973, p  < 0.01). Increased chlorophyll levels in SF cells may be a cellular adjustment to compensate for insufficient light conditions.

### INST-MFA of photoautotrophic metabolism

**Fig. 2:**
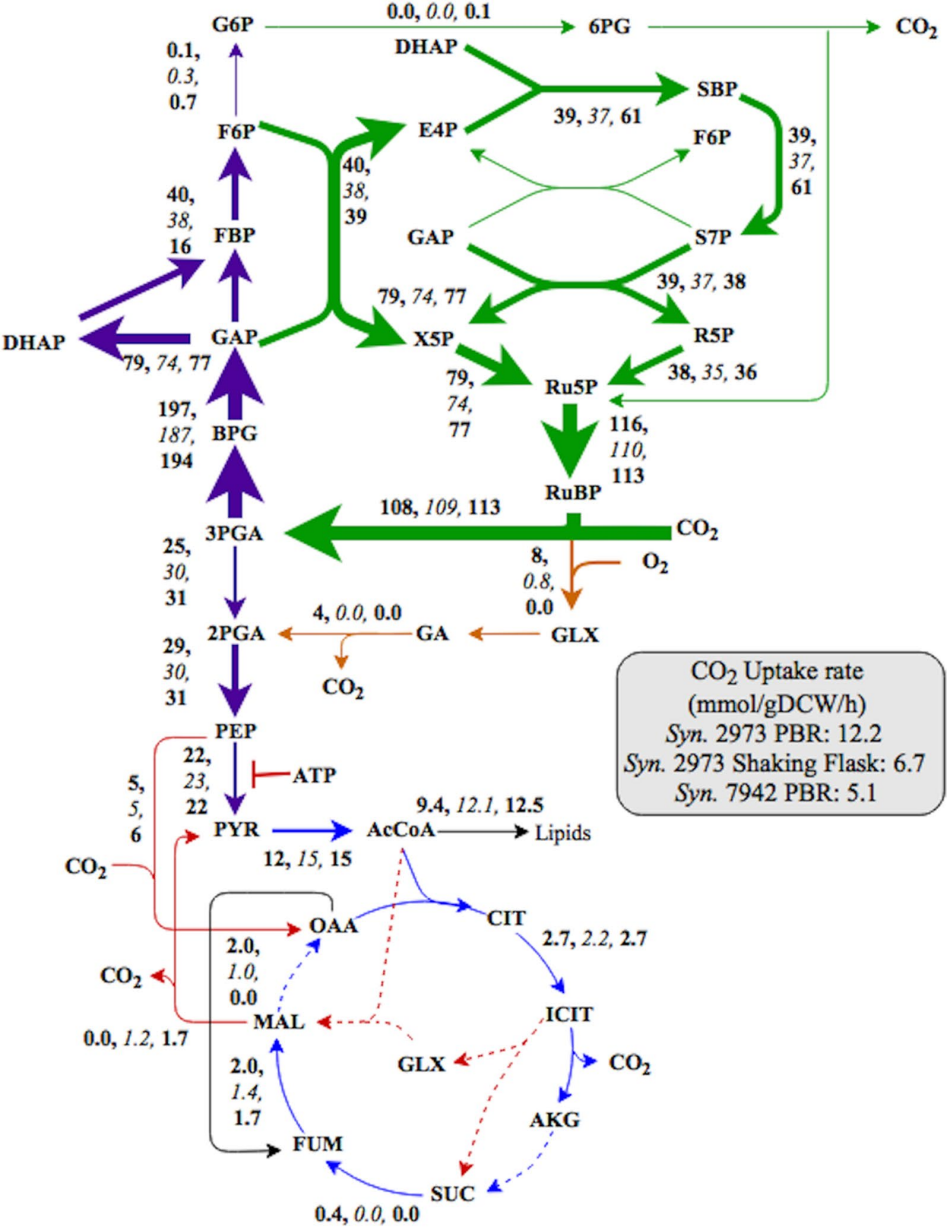
*Synechococcus* 2973 flux map determined in PBRs and SF and *Synechococcus* 7942 from the PBR. *Syn.* 2973 PBR values are listed first, followed by SF in italicized values and the third values are *Syn.* 7942 PBR. Net fluxes are normalized to net uptake of 100 mol CO_2_. Mean flux values and 95% confidence intervals are given in Additional file 1: Tables S3–S5. Estimated net CO_2_ uptake rates (mmol/gDCW/h) were 12.2 (PBR), 6.7 (SF), and 5.1 (PBR- 7942). Dotted lines represented pathways with enzymes that are not annotated for *Synechococcus elongatus* on the KEGG Genome database. **˫** indicates the inhibition of pyruvate kinase by ATP

INST-MFA relies on measurement of transient labeling data of central metabolites after a ^13^C-pulse. To quench metabolism, we used ice-cold media and liquid N_2_ bath to quickly freeze time-course samples. The approach avoided a traditional cold methanol quenching strategy that causes significant metabolite leakage for gram-negative bacteria (i.e., cyanobacteria) [12] and resulted in improved LC–MS peak quality (Additional file 1: Figure S2a, b) [13]. The INCA software package (isotopomer network compartmental analysis) [8] was used to calculate flux values (Fig. 2) based on labeling data and the metabolic network (Additional file 1: Tables S1, S2). The fitted model for the *Synechococcus* 2973 in the PBR was statistically acceptable [Sum of square residuals SSR = 713, with an accepted range of (597 740)], whereas the best fit of the SF model could not be completely explained by measurement errors (SSR = 981, with an accepted range of [382 471]). The fitted model for *Synechococcus* 7942 in the PBR was statistically acceptable (SSR = 616, range of [543 679]). Biomass composition information and CO_2_ uptake rate were used to improve the accuracy of the flux maps. Flux-partitioning across conditions and strains was compared through relative flux values that were normalized to CO_2_ uptake rate and are plotted in Fig. 2 (Confidence intervals and dilution factors are described in Additional file 1: Tables S3, S4, S5; Additional file 1: Figures S3, S4 show isotopomer fitting quality for individual metabolites). Additionally, labeling dynamics for citrate and malate are compared between *Synechococcus* 2973 and 7942 in Fig. 5. ^13^C-enrichment for key metabolites is presented in Fig. 5; Additional file 1: Figures S5, S6. The slower labeling pattern of certain metabolites may be indicative of the presence of metabolically inactive pools in bulk cytoplasm. To account for this problem, INST-MFA has employed dilution factors [3]. The isotopic dilutions in certain metabolites during non-stationary labeling suggest a non-homogeneous intracellular environment.

### *Synechococcus* flux maps in PBR and SF cultures

Figure 2 provides a comparison of the flux distributions between *Synechococcus* 2973 PBR and SF cultures and *Synechococcus* 7942 PBR cultures. Although the cells have significantly different CO_2_ fixation rates and biosynthesis fluxes (i.e., *Synechococcus* 2973 PBR cultures contained more protein synthesis fluxes, while *Synechococcus* 2973 SF and *Synechococcus* 7942 PBR cultures had higher carbon allocation to glycogen), the flux ratios in central metabolism (the Calvin cycle and the TCA pathways) were similar after normalization to CO_2_ uptake rate. Regardless of the strain and growth rate, *Synechococcus* did not exhibit flux through OPPP, and approximately 12–14% of the fixed carbon was directed to the TCA cycle similar to another recent study of *Synechococcus* 7942 [14]. Here, *Synechococcus* had no flux from *a*KG to FUM, resulting in a linear TCA pathway that is consistent with those in leaves or other photosynthetic tissues [3, 4, 15]. The flux distribution explained the absence of a number of key genes in *Synechococcus* 2973 genome, including succinyl-CoA ligase, malate dehydrogenase, α-ketoglutarate dehydrogenase, and GABA shunt genes. Although cyanobacteria are thought to employ a cyclic TCA cycle via GABA shunt for respiratory energy production [7], a complete TCA cycle seems non-essential for the *Synechococcus* strains possibly due to evolutionary non-necessity or evolved energy balancing [16]. Based on genome annotation, *Synechococcus* synthesizes fumarate from aspartate via purine metabolism, which can then be converted into malate and succinate (dynamic labeling data shown in Additional file 1: Figure S5) providing a possible source of several TCA organic acids. *Synechococcus* 2973 had significant anaplerotic fluxes through PEP carboxylase for the synthesis of TCA cycle intermediates, but reduced flux through the malic enzyme (less than ~ 1% of total fixed CO_2_ in both the PBR and SF conditions). Among tested culture conditions, there were noticeable differences in respiratory fluxes. Specifically, photorespiration in *Synechococcus* 2973 was elevated in the PBR condition possibly to rebalance carbon and dissipate excessive reducing equivalents generated by high light. In contrast, *Synechococcus* 7942 demonstrated lower photorespiration activity under PBR conditions.

### Isotopic metabolite profiling for relative pool size estimation

**Fig. 3:**
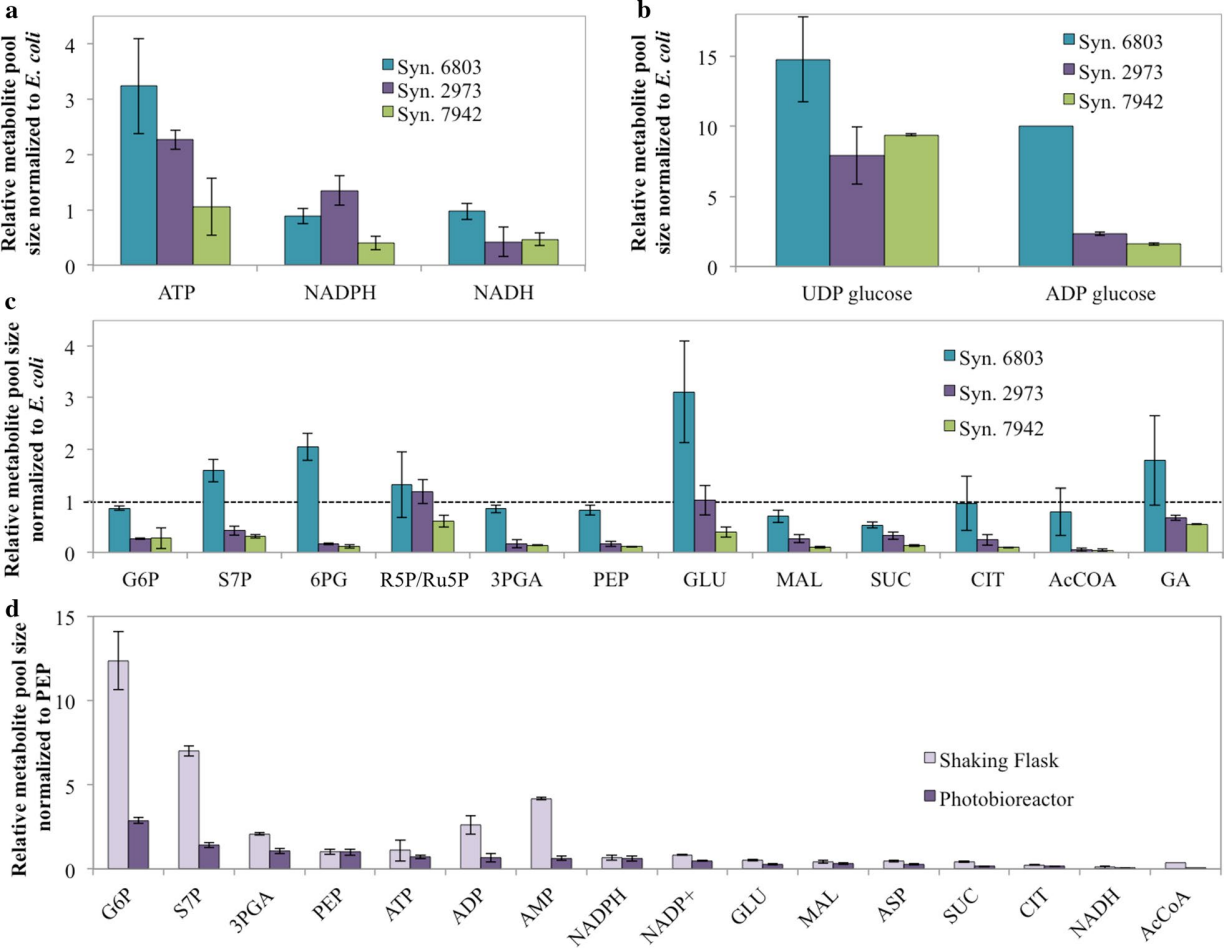
Relative pool sizes of *Synechococcus* 2973, *Synechococcus* 7942, and *Synechocystis* 6803 to *E.coli* K-12 under continuous light PBR conditions. A ratio of 1 indicates the same metabolite concentrations (normalized to gram biomass) between cyanobacteria and *Escherichia coli*. A ratio greater than 1 indicates a larger pool size in cyanobacteria strain than *Escherichia coli*. Standard deviations are based on three cyanobacteria biological replicates. **a** Average relative pool size of energy molecules compared to *E. coli* in PBR conditions. **b** Average relative pool size of UDP glucose to *E. coli* in PBR conditions. ADP glucose ratio is relative to *Synechococcus* 6803 (normalized to 10) due to the lack of ADP glucose in the *E. coli* control. **c** Relative metabolite pool size of *Synechococcus* 2973, *Synechococcus* 7942 and *Synechocystis* 6803 in PBR conditions to *E. coli* K-12. **d** Relative metabolite pool size normalized to free glutamate between the shaking flask and PBR conditions for *Synechococcus* 2973

We analyzed the change of metabolite concentrations in different cyanobacterial cultures under continuous light PBR conditions [17]. Metabolite leakage for gram negative cyanobacterial cells makes precise determination of cyanobacterial pool sizes difficult; therefore, we used isotopic ratio method to benchmark the change of cyanobacterial metabolite pools against intracellular metabolites from a standard generated from *E. coli*. Figure 3 shows the relative ratio of metabolite pool sizes normalized by the amount of biomass. Compared to *E. coli*, cyanobacterial R5P/Ru5P and other sugar phosphates were abundant, whereas the pool sizes of TCA metabolites were significantly low (Fig. 3c). *Synechococcus* 2973 had generally smaller metabolite pools than *Synechocystis* 6803 (with the exception of R5P/Ru5P) and similar metabolite pools compared to *Synechococcus* 7942 (Fig. 3c) with the exception of UDP glucose (i.e., *Synechococcus* 7942 contained more UDP glucose for carbohydrate synthesis) (Fig. 3b). Notably, *Synechococcus* 2973 had the highest NADPH concentration among the three cyanobacterial species (Fig. 3a). In addition, *Synechococcus* 2973 PBR cultivations had decreased central metabolite concentrations (mainly sugar phosphates) relative to SF cultures (Fig. 3d). Comparing two growth conditions of *Synechococcus* 2973 (Fig. 3d), the SF cells contained more ADP and AMP than PBR cells, which may suggest a change in energy charges (relative ATP concentration to overall adenosine phosphate concentrations) under suboptimal light conditions.

### Effect of exogenous organic acids on *Synechococcus* 2973’s growth

**Fig. 4:**
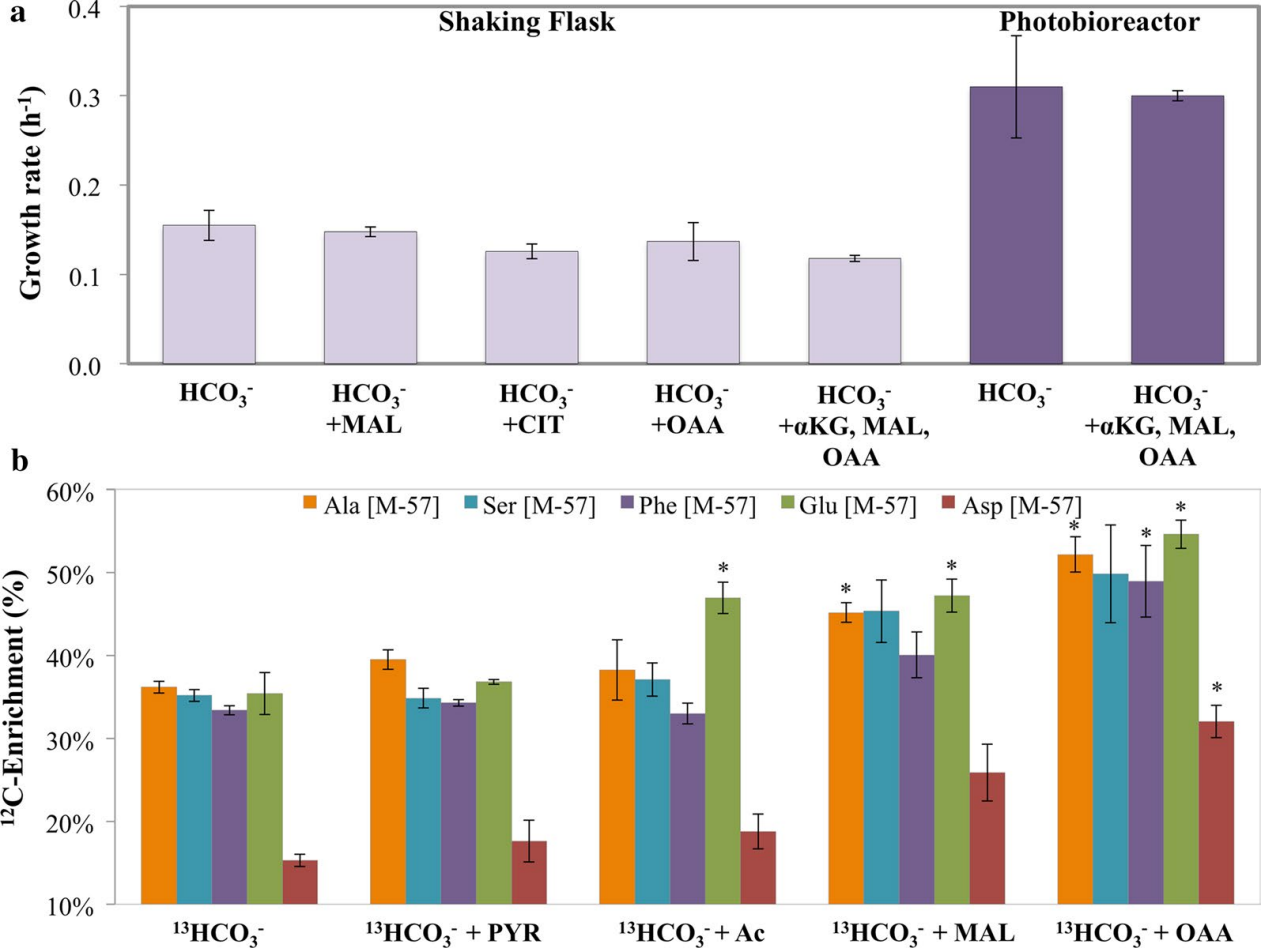
Shaking flask biomass growth of *Synechococcus* 2973 in the presence of carbon sources. **a** Growth rate (h^−1^) of *Synechococcus* 2973 in two conditions when fed with 4 g L^−1^ sodium bicarbonate and 6 mM of malate (MAL), acetate (AC), citrate (CIT), OAA, alpha-ketoglutarate (*a*KG), and a mix. Standard deviation is calculated from biological duplicates. There was no significant difference between growth rates. **b** Percent ^12^C-enrichment in amino acids through provision of 4 g L^−1^ of ^13^C-bicarbonate and 6 mM of unlabeled organic acids in SF cultures (labeling for 48 h) compared against the control of just 4 g L^−1^ of ^13^C-bicarbonate. The amino acids were chosen because of their direct relation to key metabolic precursors (Ala->PYR, Ser->3PGA, Phe->PEP/E4P, Glu->α-KG, Asp->OAA). [M-57] represents the mass signals of unlabeled amino acid (without fragmentation by GC–MS). Note: *Synechococcus* 2973 used unlabeled organic carbon sources to synthesize proteinogenic amino acids, the [M-57] % of amino acids increased compared to the control sample that was only fed with ^13^C-bicarbonate. Standard deviations are a result of biological duplicates *represents a p value of < 0.02 using two-tailed equal variance Student’s t Test

*Synechococcus* 2973 shows significant sugar phosphate interconversion, but the TCA cycle is incomplete with limited flux and reduced metabolite pools. We hypothesized that the addition of exogenous organic substrates may alleviate such biosynthesis bottlenecks; however, cell growth was not enhanced after supplying OAA, malate, αKG or organic acids (pyruvate and acetate) in either PBR or SF conditions (Fig. 4a), even though the cells demonstrated the ability to incorporate exogenous nutrients into proteinogenic amino acids (especially for OAA; Fig. 4b). Unlabeled malate from the culture medium could be significantly incorporated into aspartate, suggesting an uncharacterized malate dehydrogenase to synthesize oxaloacetate (the precursor of aspartate). In summary, *Synechococcus* 2973 shows limited capability to efficiently utilize organic acids and convert these nutrients into cyanobacteria biomass [18].

## Discussion

### Flux comparisons among cyanobacterial species

*Synechococcus* 2973 and 7942 have a number of phenotypic differences. The CO_2_ uptake rate in *Synechococcus* 2973 was twofold greater than *Synechococcus* 7942. *Synechococcus* 2973 showed up to a 3.5-fold greater ^13^C-enrichment over *Synechococcus* 7942 for metabolites such as FBP and S7P (Additional file 1: Figure S6) at 40 s, indicating greater Calvin cycle fluxes. From the normalized flux map, there are differences in the carbon partitioning as presented in Fig. 2 and Additional file 1: Figure S7. *Synechococcus* 2973 fluxes through fructose-biphosphate aldolase and phosphatase were increased while the fluxes towards glycogen and the malic enzyme were reduced. Flux from F6P to G6P, which is the key step towards OPPP and sugar storage metabolism, was significantly decreased in *Synechococcus* 2973 under both conditions. Malate and citrate were labeled rapidly in *Synechococcus* 2973 due to the enhanced PEPC (phosphoenolpyruvate carboxylase) reaction. Unlike *Synechocystis* 6803 that maintains a functional GABA shunt [7], *Synechococcus elongatus* apparently does not contain the genes to operate a complete TCA cycle (as annotated by the KEGG Genome database), but based on labeling data presented here may utilize a linear pathway from OAA to make citrate and *a*KG. The GABA shunt contains one decarboxylating reaction and the absence of this shunt reduces CO_2_ loss. A succinate dehydrogenase deletion mutant was created, and the mutant showed no growth defects under continuous light conditions, which supports the absence of fluxes from *a*KG to fumarate. Unlike *Synechocystis* 6803, *Synechococcus* 2973 pyruvate is predominately made from flux through pyruvate kinase with little flux through the malic enzyme (MAL → PYR + CO_2_). Pyruvate kinase is a key step for producing pyruvate, but its activity in some cyanobacterial species can be inhibited by high ATP/ADP ratios during photosynthesis [19]. For example, the majority of pyruvate synthesis in *Synechocystis* 6803 [3] and *Synechococcus* 7942 [14] during slow growth with suboptimal conditions was a result of malate degradation. Overexpression of pyruvate kinase for pyruvate synthesis improved cyanobacterial lactate and alcohol productions [20, 21]. *Synechococcus* 2973 has naturally evolved to overcome this pyruvate synthesis bottleneck, de-coupling pyruvate kinase inhibition from high photosynthesis rates. Furthermore, as shown in Fig. 2 and genome scale modeling [22], strong photorespiration is essential for high light photosynthesis and maximal *Synechococcus* 2973 growth despite its net carbon loss. *Synechococcus* 2973 exhibits twice the O_2_ evolution rate of *Synechococcus* 7942 [11]. Water splitting results in heightened local oxygen concentration and increased linear electron flow, both of which may increase the need for photorespiration or cyclic electron flow [23].

The OPPP can generate NADPH and oxidize sugar phosphates as a means to derive more reduced compounds but its activation at the same time as the Calvin Cycle would generate a futile cycle that reduces the efficient use of carbon and energy, which is why in higher plants the two are coordinately regulated by multiple mechanisms including redox state and pH [24, 25]. Due to lack of sufficient regulation or Calvin cycle bottlenecks, slow growing *Synechocystis* 6803 demonstrated ~ 12% CO_2_ loss through OPPP under continuous light [3], while *Synechococcus* does not exhibit significant flux through this pathway. To further test this observation, we constructed *Synechococcus* 2973 *zwf* and *pgl* deletion mutants by disrupting the gene encoding G6P dehydrogenase and 6-phosphogluconolactonase, respectively, so that G6P could not be converted to 6PG (Additional file 1: Figure S2e, f). The mutants showed similar growth rate to the wild-type strain under continuous light and diurnal bioreactor conditions (Fig. 1), indicating the OPPP did not provide a benefit to the organism and was futile for photoautotrophic metabolism in *Synechococcus* 2973 [26]. These mutants and metabolic insights from the quantification of central carbon fluxes suggest new strategies for the redirection of carbon flux for the bioengineering of photosynthetic organisms. The observation that *Synechococcus* 2973 exhibits a similar growth phenotype to *Synechococcus* 7942 under diurnal conditions emphasizes the differences that can occur as a result of changes in light provided by optimal bioreactor conditions.

### The optimal photoautotrophic metabolism (reduced pool sizes, enhanced energy levels, and repartitioning of biomass composition)

Metabolite concentrations are tied to input/output fluxes and can provide insight into biochemical level regulation [27, 28]. The lack of correlation between pool sizes and absolute fluxes has been shown in literature [15, 29], as well as here between *Synechococcus* 2973, *Synechococcus* 7942, and *Synechocystis* 6803. Compared to SF cultures and *Synechocystis* 6803, the concentrations of a number of central metabolites from glycolysis and TCA in *Synechococcus* 2973 were reduced during the PBR conditions. Less metabolite accumulation in central metabolism under PBR conditions could indicate reduced feedback or higher rates of metabolite turnover. From literature, it has been suggested that as cell growth rates and carbon fluxes increase, the fraction of anabolic enzymes is increased to pull central metabolites towards biomass synthesis and thus decreases their pool sizes [30]. Conversely, the heterotroph *E. coli* had intracellular acetyl-CoA/organic acid concentrations much higher than cyanobacteria (Fig. 3c). Such observations, together with cultivation experiments using organic acids, indicated that the TCA cycle and its associated anabolic pathways in *Synechococcus* 2973 operate at lower rates than in *E. coli*, which would limit production of chemicals from the cyanobacterial TCA cycle [4]. In *Synechococcus* 2973, the photosynthetic capacity generates sugar phosphates readily (FBP phosphatase/aldolase, fructose-bisphosphatase, transketolase, and RuBisCO reactions) that can enhance its growth [31–33] and could potentially be applied for biotechnological productions [34].

Additionally, the higher energy charge in the PBR than SF for *Synechococcus* 2973 may benefit anabolic metabolism and cell tolerance to stress conditions [35]. Cyanobacterial biosynthesis has been closely tied to photosynthesis and changing bioreactor conditions [36]. In SF cultures, there was an increase in lower energy molecules (NADP^+^, ADP, and AMP) inhibiting anabolism, possibly resulting in higher levels of some central metabolites. The PBR grown *Synechococcus* 2973 showed the highest NADPH level that may reflect enhanced light-harvesting that can facilitate organic carbon assimilation and biomass growth [20, 37]. *Synechococcus* 2973 adjusts its PSII/PSI ratio throughout growth although it was found to have a slightly lower PSII/PSI ratio than *Synechococcus* 7942 while exhibiting greater O_2_ evolution [11]. Additionally, it may have a more optimal ATP/NADPH ratio to sustain metabolism while not exceeding ATP demand [22], which may be important to further unlocking cyanobacteria growth constraints. While CO_2_ fixation rate is a key factor for differences in phenotypes, CO_2_ fixation rate is influenced by optimal energy balance, biomass distribution/central carbon fluxes, and compartmentalization of photosynthetic reactions.

*Synechococcus* 2973 PBR cells produce little glycogen (8 ~ 10 times less than PBR cells of *Synechococcus* 7942 and *Synechocystis* 6803 [38]). When less carbon is allocated to make glycogen, more can be partitioned toward synthesis of biomass, resulting in greater protein levels in *Synechococcus* 2973 than 7942. Consequently, *Synechococcus* 2973 may have more photosystems/RuBisCO proteins for effective photosynthesis. In contrast, *Synechococcus* 2973 produced significantly more glycogen under suboptimal SF conditions, leading to longer cell doubling times.

### Metabolic features of *Synechococcus* 2973 for bio-production applications

Understanding and exploring native pathways with high metabolic strengths is a promising direction for future microbial cell factories [39]. Our flux results indicate that *Synechococcus* 2973 is advantageous for the synthesis of targeted products from sugar phosphate pathways under optimal bioreactor conditions. Recently, researchers have engineered *Synechococcus* 2973 to produce ~ 9 g L^−1^ of sucrose under potassium chloride stress [40]. This engineered strain also demonstrated potential to produce valuable polysaccharide products. It has been observed that while cyanobacteria have a flexible photosynthetic metabolism, there are a high number of essential metabolic genes involved in photoautotroph growth. This presents additional challenges for mutant strain generation or extensive genetic modification, and the knowledge gained from this study will help surmount the challenges facing cyanobacterial photo-biorefineries [41].

### Subpopulations, metabolic inactive pools, and substrate channeling

Modeling results indicated the presence of unlabeled pools that required additional dilution factors for isotopic data fitting. For SF cultures, an increase in dilution factors was necessary to improve fitting, still resulting in an unaccepted SF fit. The dilution of the metabolite active pool by unlabeled metabolites infers phenotypic heterogeneity, i.e., the presence of inactive/non-growth cells or the presence of pre-existing sources of carbon that are more slowly turned over after ^13^C-bicarbonate pulses [42]. Inactive cells would not be involved in significant photosynthesis within the duration of the labeling period and may induce reflux of unlabeled carbon into the metabolic network [43]. Inactive subpopulations can be increased by poor light transmittance due to suboptimal culture conditions, cell self-shading and/or insufficient mixing conditions [44]. This may explain why *Synechococcus* 2973 does not exhibit significant growth advantages in SF conditions.

**Fig. 5:**
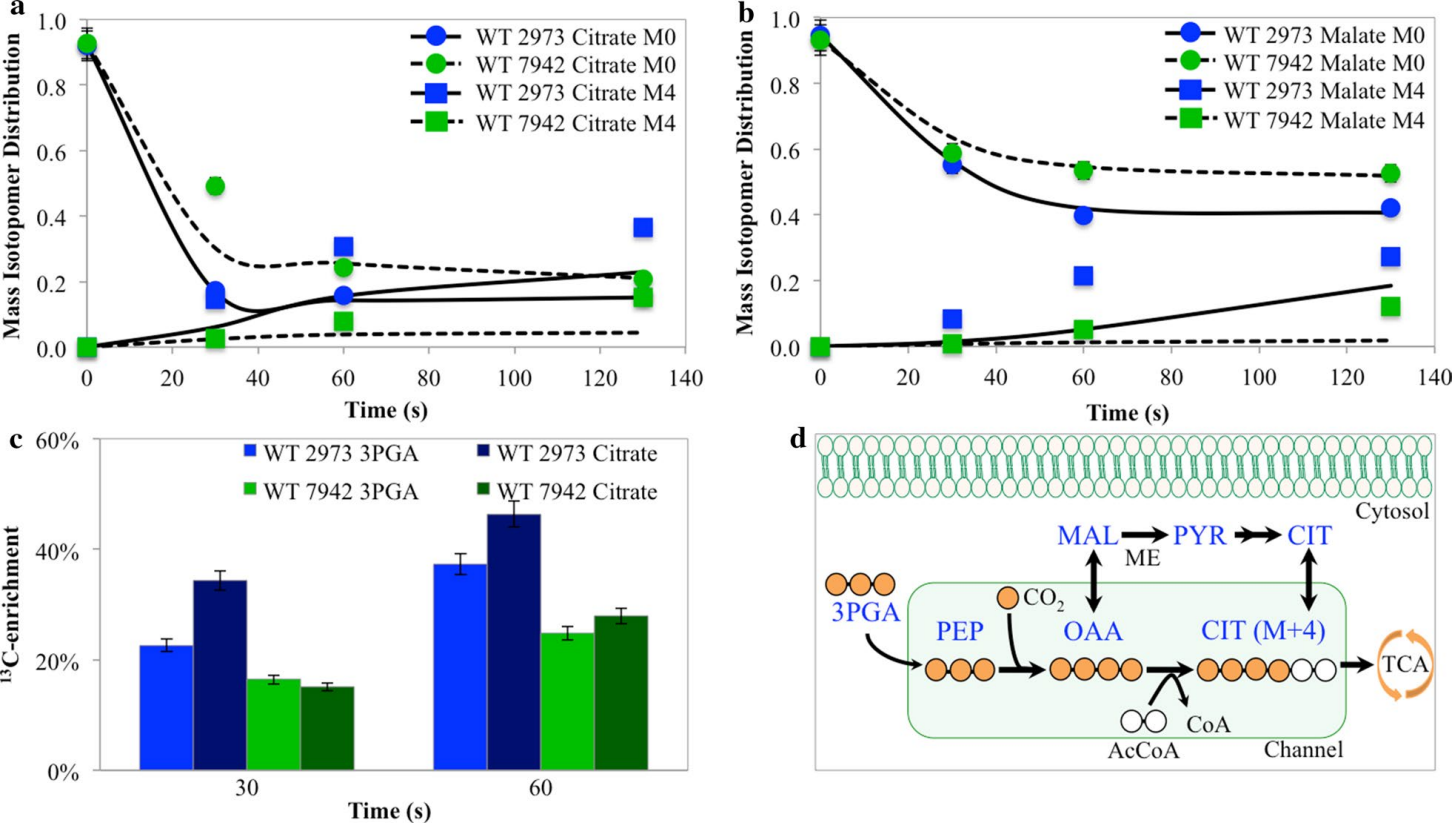
Labeling dynamics for *Synechococcus* 2973 and 7942 in photobioreactor conditions for Citrate, 3PGA, and Malate. **a** The mass isotopomer distribution for citrate (M+0) and (M+4) fractions as a function of time. Experimentally measured MIDs are the data points, while the line represents the fitted data from INCA. **b** The mass isotopomer distribution for malate (M+0) and (M+4) fractions as a function of time. Experimentally measured MIDs are the data points, while the line represents the fitted data from INCA. **c** The total ^13^C-enrichment of 3PGA compared to citrate as a function of time from experimentally measured MIDs. **d** Substrate channel scheme proposed based on labeling data from *Synechococcus* 2973. Experimentally measured MIDs with error bars represent standard deviations from biological duplicates

Metabolite channeling could also contribute to the observed phenotype. Channels pass metabolite intermediates between enzymes without intracellular diffusion. Channeling increases pathway efficiency [45] and is often associated with microcompartments (i.e., carboxysomes) or enzyme proximity [45–47]. For fast growing *E. coli* species, glycolysis channeling has been evident [48]. Substrate channeling can be inferred from transient labeling experiments [45]. When tracing ^13^C-enrichment from 3PGA to downstream metabolites in *Synechocystis* 6803, it was previously found that certain downstream metabolites could be labeled faster than precursors (e.g., PEP was labeled faster than 3PGA) [3, 29]. In this study, we observed ^13^C-enrichment might not completely follow expected precursor-product relationships (Additional file 1: Figure S6). Comparing to *Synechococcus* 7942, *Synechococcus* 2973 showed rapid ^13^C-enrichment in S7P over precursors. This can be explained by possible channels in the Calvin cycle that may influence the labeling of certain metabolites and provide advantages for CO_2_ fixation. *Synechococcus* 2973 also shows evidence for channeling of glycolysis intermediates towards the TCA pathway with faster citrate M+4 distribution over time that exceeds the ^13^C-enrichment levels of 3PGA (Fig. 5). However, whether differences are a result of traditional channeling definitions or represent the presence of multiple pools separated through additional spatial compartments cannot be conclusively determined from our studies. These observations suggest a heterogenetic distribution of intracellular metabolites as well as possible spatial organization of enzymes to confer growth benefits.

## Conclusion

*Synechococcus* 2973 demonstrates high photosynthesis, efficient carbon fixation and small fluxes towards carbon loss and transient storage pathways under optimal growth conditions. These metabolic traits suggest this strain may be a promising platform to produce high-value compounds in well-controlled PBR conditions. Under suboptimal conditions, *Synechococcus* 2973 has inferior photosynthesis and decreased biomass synthesis, causing an accumulation of central carbon metabolites. Moreover, *Synechococcus* strains with different growth rates maintain similar flux distributions in central pathways. These observations offer insight into flux response to adaptive evolution. From a technological perspective, INST-MFA of cyanobacteria is not only important for genome-to-phenome mapping but also crucial for the rational application of platform photo-biorefineries. Currently, INST-MFA is still challenging, and flux results may be influenced by substrate channeling or subpopulations. Sorting out these factors will require advanced labeling experiments [49–52] to complement computer modeling and labeling experiments. Therefore, mutant creations and biomass composition analysis were performed to offer information resources to aid this study.

## Methods

### Cultivation conditions, and transient labeling experiments

*Synechococcus* 2973 was grown in BG-11 medium (pH 8.0–8.5) at 38 °C with 3% CO_2_ aeration (2000 mL min^−1^) and continuous 500 μmol photons/m^2^s under photobioreactors (Additional file 1: photo 1). *Synechococcus* 2973 was also grown in shaking flasks (atmospheric CO_2_, 38 °C and continuous and diurnal (12 h intervals) 100 μmol photons/m^2^s light, 250 rpm). *Synechococcus* 7942 was grown in identical conditions, except in its optimal PBR conditions, where the light condition was 300 μmol photons/m^2^s. Higher light irradiance inhibits *Synechococcus* 7942 growth [2]. For the isotopic pool-size experiment, *Synechocystis* 6803 was grown in identical photobioreactors at 30 °C, 3% CO_2_ aeration and continuous 300 μmol photons/m^2^s light. For each labeling experiment, cultures were diluted to an OD_730_ 0.05 in BG-11 medium without citrate (so citrate could not be used a carbon source or affect bulk citrate labeling dynamics) and grown to OD_730_ 0.6 (exponential phase) before the experiment was initiated. Prior to the experiment, the 3% CO_2_ aeration was replaced with air (0.04% CO_2_) and a 2-mL aliquot of saturated NaH^13^CO_3_ (> 98% purity, Sigma Aldrich, St Louis) was injected into each PBR or shaking flask (SF) for a final concentration of 4 g L^−1^ of NaH^13^CO_3_ to saturate the cell with CO_2_. The use of ^13^C-bicarbonate rather than ^13^CO_2_ is sufficient because ^13^C-bicarbonate quickly equilibrates within the media without gas–liquid mass transfer limitations.

After the ^13^C-pulse, each identical culture in the PBR and SF was quenched at different time intervals (20, 30, 40, 60, 90, 150, 300, 600, 1200, 3600, 7200 s). These time-courses samples were analyzed to capture labeling dynamics of metabolites. Timed biological duplicates were used to generated standard deviations for experimentally measured MID values; however, a minimum standard deviation of 3% was used in INCA model.

### Metabolite quenching, extraction, and analysis

Fast-cooling via cold solvents followed by centrifugation is reliable for recovering intracellular labeled metabolites [13]. After a ^13^C-pulse, samples were quenched by mixing with ice-cold minimal medium (< −5 °C, without a carbon source) in a falcon tube, which was immediately bathed in liquid N_2_ to sustain cold temperature (To avoid ice formation, the culture sample was rapidly swirled for several seconds). Then the quenched biomass samples (< 0 °C) were harvested via refrigerator centrifuge (3 min, 6000 rpm). For LC–MS analysis, the pelleted samples were extracted with a chloroform–methanol method [15]. Ion-pairing LC–MS/MS was performed at the Proteomics and Mass Spectrometry Facility, Donald Danforth Plant Science Center, St. Louis (details in Additional file 1: Part 1). The labeling of energy molecules (e.g., NADH and ATP), organic acids and acetyl-CoA were quantified using hydrophilic interaction liquid chromatography (HILIC) coupled to electrospray time-of-flight MS [53] at Joint Bio-Energy Institute, CA.

### Estimation of relative pool sizes of metabolites

Changes of metabolite pool sizes in cyanobacteria were analyzed by an MS isotopomer ratio approach [54] (using fully labeled cell extracts as internal standards for semi-quantitative metabolomics [55]). Specifically, *E. coli* K-12 was cultured with uniformly labeled ^13^C-glucose and ^13^C-sodium bicarbonate in an M9 minimal media. The estimation of metabolite pool sizes was performed by mixing a known amount of labeled *E. coli* biomass with unlabeled cyanobacteria cultures (three biological replicates). Then, the mixtures were quenched by a liquid N_2_ bath and extracted for HILIC-MS analysis (Additional file 1: Figure S2c). The isotopic ratio of each metabolite (labeled vs. unlabeled) was normalized by the amount of *E. coli* and cyanobacterial biomass, respectively.

### Biomass composition analysis

The cells were harvested during exponential growth by centrifugation, and cell pellets were washed with 0.9% NaCl and ddH_2_O and then freeze-dried. Protein and amino acid compositional analysis was performed by the Molecular Structure Facility, University of California (Davis, CA). Carbohydrates, lipids, chlorophyll *a*, and ash were measured by previously reported methods (details in Additional file 1: Part 1).

### GC–MS analysis of proteinogenic amino acids

To confirm the capability of *Synechococcus* 2973 to uptake organic acids, unlabeled seed cultures were inoculated (4% inoculation ratio) into the medium with fully labeled NaH^13^CO_3_ and unlabeled substrates (6 mM) including acetate, TCA cycle intermediates (e.g., malate and citrate), and pyruvate in shaking flasks or PBRs for cultivation of 48 h. The labeling in proteinogenic amino acids was then analyzed using a TBDMS method [56].

### Isotopically nonstationary MFA

MFA is estimated based on isotope labeling dynamics of the free metabolites. The MFA model included the Calvin cycle, pentose phosphate pathway, TCA cycle, the glyoxylate shunt, anaplerotic pathways, and photorespiration pathway. A list of reactions in the network model with their atom transitions is provided in Additional file 1: Table S1. A lumped biomass equation based on biomass composition analysis and biochemical equations was used to fit the model to biomass production (Additional file 1: Table S2). The INCA platform [8] used a custom MATLAB ODE solver to fit over 890, 600, and 800 individual mass fragments for *Synechococcus* 2973 PBR, SF, and *Synechococcus* 7942 PBR to obtain fluxes in the assumed cyanobacterial network, respectively. INCA provided a goodness of fit via a Chi square statistical test as well as confidence intervals of all estimated parameters (Model formulation details are included in the Additional file 1: Part 1).

### Construction of the *zwf, pgl,* and the succinate dehydrogenase deletion mutants

Three mutants were built, which served to independently validate the related aspects of metabolism described by the modeling process. The ***∆*** *zwf* mutant was constructed by insertional mutagenesis of the first enzyme of the OPPP, glucose-6-phosphate 1 dehydrogenase. The ***∆*** *zwf* mutant was characterized via growth rate (Fig. 1). The mutant activity was confirmed via IP-LC–MS/MS by the absence of the downstream product of its disrupted gene, 6-phosphogluconate (Additional file 1: Figure S2d–f). The *∆pgl and ∆sdh* mutant was constructed by a *cpf1* based CRISPR system was used to delete succinate dehydrogenase from start codon to stop codon from the chromosome (Additional file 1: Part 1).

## Additional file

**Additional file 1.** Deciphering cyanobacterial phenotypes for fast photoautotrophic growth via isotopically nonstationary metabolic flux analysis: Supporting Information. Supporting information contains biomass measurements, model development, Supporting Tables 1–5, Supporting Figures 1–7, and a Supporting photo.

## Abbreviations

2PG: 2-phosphoglycolate
2PGA: 2-phosphoglycerate
3PGA: 3-phosphoglycerate
6PG: 6-phosphogluconate
Ac: acetate
AcCoA: acetyl-CoA
ADP: adenosine diphosphate
ADP glucose: adenosine diphosphoglucose
αKG: alpha-ketoglutarate
AMP: adenosine monophosphate
ATP: adenosine triphosphate
BPG: 1,3-bisphosphoglyceric acid
CIT: citrate
DHAP: dihydroxyacetone phosphate
E4P: erythrose 4-phosphate
F6P: fructose 6-phosphate
FBP: fructose 1,6-diphosphate
FUM: fumarate
G1P: glucose 1-phosphate
G6P: glucose 6-phosphate
GA: glycerate
GABA: gamma-aminobutyric acid
GAP: glyceraldehyde 3-phosphate
GLC: glycolate
GLX: glyoxylate
GLYC: glycogen
ICIT: isocitrate
MAL: malate
MeOH: methanol
MID: mass isotopomer distribution
NAD^+^: nitcotinamide adenine dinucleotide
NADH: nitcotinamide adenine dinucleotide (reduced)
NADP^+^: nitcotinamide adenine dinucleotide phosphate
NADPH: nitcotinamide adenine dinucleotide phosphate (reduced)
OAA: oxaloacetate
OPPP: oxidative pentose phosphate pathway
PEP: phosphoenol pyruvate
PYR: pyruvate
R5P: ribose 5-phosphate
Ru5P: ribulose 5-phosphate
RuBP: ribulose 1,5-diphosphate
S7P: sedoheptulose 7-phosphate
SBP: sedoheptulose 1,7-diphosphate
SUC: succinate
TCA: tricarboxylic acid cycle
UDP: glucose uridine diphosphoglucose
X5P: xylulose 5-phosphate

## References

[1] Microalgae Sayre R (2010). The potential for carbon capture. Bioscience.

[2] Yu J, Liberton M, Cliften PF, Head RD, Jacobs JM, Smith RD, Koppenaal DW, Brand JJ, Pakrasi HB (2015). *Synechococcus elongatus* UTEX 2973, a fast growing cyanobacterial chassis for biosynthesis using light and CO_2_. Sci Rep.

[3] Young JD, Shastri AA, Stephanopoulos G, Morgan JA (2011). Mapping photoautotrophic metabolism with isotopically nonstationary ^13^C flux analysis. Metab Eng.

[4] Xiong W, Morgan JA, Ungerer J, Wang B, Maness PC, Yu J (2015). The plasticity of cyanobacterial metabolism supports direct CO_2_ conversion to ethylene. Nat Plants.

[5] Yang C, Hua Q, Shimizu K (2000). Energetics and carbon metabolism during growth of microalgal cells under photoautotrophic, mixotrophic and cyclic light-autotrophic/dark-heterotrophic conditions. Biochem Eng J.

[6] You L, Berla B, He L, Pakrasi HB, Tang YJ (2014). ^13^C-MFA delineates the photomixotrophic metabolism of *Synechocystis* sp. PCC 6803 under light-and carbon-sufficient conditions. Biotechnol J.

[7] Xiong W, Brune D, Vermaas WFJ (2014). The γ-aminobutyric acid shunt contributes to closing the tricarboxylic acid cycle in *S ynechocystis* sp. PCC 6803: the γ-aminobutyric acid shunt in *Synechocystis*. Mol Microbiol.

[8] Young JD (2014). INCA: a computational platform for isotopically non-stationary metabolic flux analysis. Bioinformatics.

[9] Yuan J, Bennett BD, Rabinowitz JD (2008). Kinetic flux profiling for quantitation of cellular metabolic fluxes. Nat Protoc.

[10] Pramanik J, Keasling JD (1997). Stoichiometric model of *Escherichia coli* metabolism: incorporation of growth-rate dependent biomass composition and mechanistic energy requirements. Biotechnol Bioeng.

[11] Mueller TJ, Ungerer J, Pakrasi HB, Maranas CD (2017). Identifying the metabolic differeneces of a fast-growth phenotype in *Synechococcus* UTEX 2973. Sci Rep.

[12] Bolten CJ, Kiefer P, Letisse F, Portais J-C, Wittmann C (2007). Sampling for metabolome analysis of microorganisms. Anal Chem.

[13] Millard P, Massou S, Wittmann C, Portais J-C, Létisse F (2014). Sampling of intracellular metabolites for stationary and non-stationary ^13^C metabolic flux analysis in *Escherichia coli*. Anal Biochem.

[14] Jazmin LJ, Xu Y, Cheah YE, Adebiyi AO, Johnson CH, Young JD (2017). Isotopically nonstationary ^13^C flux analysis of cyanobacterial isobutyraldehyde production. Metab Eng.

[15] Ma F, Jazmin LJ, Young JD, Allen DK (2014). Isotopically nonstationary ^13^C flux analysis of changes in *Arabidopsis thaliana* leaf metabolism due to high light acclimation. Proc Natl Acad Sci.

[16] Rubin BE, Wetmore KM, Price MN, Diamond S, Shultzaberger RK, Lowe LC, Curtin G, Arkin AP, Deutschbauer A, Golden SS (2015). The essential gene set of a photosynthetic organism. Proc Natl Acad Sci.

[17] Bennett BD, Kimball EH, Gao M, Osterhout R, van Dien SJ, Rabinowitz JD (2009). Absolute metabolite concentrations and implied enzyme active site occupancy in *Escherichia coli*. Nat Chem Biol.

[18] Yan R, Zhu D, Zhang Z, Zeng Q, Chu J (2011). Carbon metabolism and energy conversion of *Synechococcus* sp. PCC 7942 under mixotrophic conditions: comparison with photoautotrophic condition. J Appl Phycol.

[19] Bricker TM, Zhang S, Laborde SM, Mayer PR, Frankel LK, Moroney JV (2004). The Malic enzyme is required for optimal photoautotrophic growth of *Synechocystis* sp. strain PCC 6803 under continuous light but not under a diurnal light regimen. J Bacteriol.

[20] Oliver JWK, Atsumi S (2015). A carbon sink pathway increases carbon productivity in cyanobacteria. Metab Eng.

[21] Angermayr SA, van der Woude AD, Correddu D, Vreugdenhil A, Verrone V, Hellingwerf KJ (2014). Exploring metabolic engineering design principles for the photosynthetic production of lactic acid by *Synechocystis* sp. PCC 6803. Biotechnol Biofuels.

[22] Nogales J, Gudmundsson S, Knight EM, Palsson BO, Thiele I (2012). Detailing the optimality of photosynthesis in cyanobacteria through systems biology analysis. Proc Natl Acad Sci.

[23] Kramer DM, Evans JR (2011). The importance of energy balance in improving photosynthetic productivity. Plant Physiol.

[24] Buchanan BB (1980). Role of light in the regulation of chloroplast enzymes. Annu Rev Plant Physiol.

[25] Werdan K, Heldt HW, Milovancev M (1975). The role of pH in the regulation of carbon fixation in the chloroplast stroma. Studies on CO_2_ fixation in the light and dark. Biochim Biophys Acta.

[26] Scanlan DJ, Sundaram S, Newman J, Mann NH, Carr NG (1995). Characterization of a *zwf* mutant of *Synechococcus* sp. strain PCC 7942. J Bacteriol.

[27] Schwender J, Hebbelmann I, Heinzel N, Hildebrandt T, Rogers A, Naik D, Klapperstuck M, Braun HP, Schreiber F, Denolf P (2015). Quantitative multilevel analysis of central metabolism in developing oilseeds of oilseed rape during in vitro culture. Plant Physiol.

[28] Buescher JM, Antoniewicz MR, Boros LG, Burgess SC, Brunengraber H, Clish CB, DeBerardinis RJ, Feron O, Frezza C, Ghesquiere B (2015). A roadmap for interpreting ^13^C metabolite labeling patterns from cells. Curr Opin Biotechnol.

[29] Huege J, Goetze J, Schwarz D, Bauwe H, Hagemann M, Kopka J (2011). Modulation of the major paths of carbon in photorespiratory mutants of *Synechocystis*. PLoS ONE.

[30] You C, Okano H, Hui S, Zhang Z, Kim M, Gunderson CW, Wang YP, Lenz P, Yan D, Hwa T (2013). Coordination of bacterial proteome with metabolism by cyclic AMP signalling. Nature.

[31] Liang F, Lindblad P (2016). Effects of overexpressing photosynthetic carbon flux control enzymes in the cyanobacterium *Synechocystis* PCC 6803. Metab Eng.

[32] Uematsu K, Suzuki N, Iwamae T, Inui M, Yukawa H (2012). Increased fructose 1,6-bisphosphate aldolase in plastids enhances growth and photosynthesis of tobacco plants. J Exp Bot.

[33] Bernstein HC, McClure RS, Hill EA, Markillie LM, Chrisler WB, Romine MF, McDermott JE, Posewitz MC, Bryant DA, Konopka AE (2016). Unlocking the constraints of cyanobacterial productivity: acclimations enabling ultrafast growth. mBio.

[34] Markou G, Nerantzis E (2013). Microalgae for high-value compounds and biofuels production: a review with focus on cultivation under stress conditions. Biotechnol Adv.

[35] Srivastava AK, Rai AN, Neilan BA (2013). Stress biology of cyanobacteria: molecular mechanisms to cellular responses.

[36] Carrieri D, Broadbent C, Carruth D, Paddock T, Ungerer J, Maness PC, Ghirardi M, Yu J (2015). Enhancing photo-catalytic production of organic acids in the cyanobacterium *Synechocystis* sp. PCC 6803 Δ*glgC*, a strain incapable of glycogen storage. Microb Biotechnol.

[37] Zhou J, Zhang F, Meng H, Zhang Y, Li Y (2016). Introducing extra NADPH consumption ability significantly increases the photosynthetic efficiency and biomass production of cyanobacteria. Metab Eng.

[38] Iijima H, Nakaya Y, Kuwahara A, Hirai MY, Osanai T. Seawater cultivation of freshwater cyanobacterium *Synechocystis* sp. PCC 6803 drastically alters amino acid composition and glycogen metabolism. Front Microbiol. 2015;6:326. 10.3389/fmicb.2015.00326 PMC4406197 25954257

[39] Nielsen J, Keasling JD (2016). Engineering cellular metabolism. Cell.

[40] Song K, Tan X, Liang Y, Lu X (2016). The potential of *Synechococcus elongatus* UTEX 2973 for sugar feedstock production. Appl Microbiol Biotechnol.

[41] Abed RM, Dobretsov S, Sudesh K (2009). Applications of cyanobacteria in biotechnology. J Appl Microbiol.

[42] Mohr W, Vagner T, Kuypers MMM, Ackermann M, LaRoche J (2013). Resolution of conflicting signals at the single-cell level in the regulation of cyanobacterial photosynthesis and nitrogen fixation. PLoS ONE.

[43] Nargund S, Misra A, Zhang X, Coleman GD, Sriram G (2014). Flux and reflux: metabolite reflux in plant suspension cells and its implications for isotope-assisted metabolic flux analysis. Mol BioSyst.

[44] He L, Wu SG, Wan N, Reding AC, Tang YJ (2015). Simulating cyanobacterial phenotypes by integrating flux balance analysis, kinetics, and a light distribution function. Microb Cell Factories.

[45] Wheeldon I, Minteer SD, Banta S, Barton SC, Atanassov P, Sigman M (2016). Substrate channelling as an approach to cascade reactions. Nat Chem.

[46] Conrado RJ, Varner JD, DeLisa MP (2008). Engineering the spatial organization of metabolic enzymes: mimicking nature’s synergy. Curr Opin Biotechnol.

[47] Jandt U, You C, Zhang YHP, Zeng AP, Zeng AP (2013). Compartmentalization and metabolic channeling for multienzymatic biosynthesis: practical strategies and modeling approaches. Fundamentals and application of new bioproduction systems, advances in biochemical engineering/biotechnology.

[48] Shearer G, Lee JC, Koo J, Kohl DH (2005). Quantitative estimation of channeling from early glycolytic intermediates to CO_2_ in intact *Escherichia coli*. FEBS J.

[49] Allen DK, Laclair RW, Ohlrogge JB, Shachar-Hill Y (2012). Isotope labelling of Rubisco subunits provides in vivo information on subcellular biosynthesis and exchange of amino acids between compartments. Plant Cell Environ.

[50] Mandy DE, Goldford JE, Yang H, Allen DK, Libourel IGL (2014). Metabolic flux analysis using ^13^C peptide label measurements. Plant J.

[51] Allen DK, Goldford J, Gierse JK, Mandy D, Diepenbrock C, Libourel IG (2014). Quantification of peptide *m/z* distributions from ^13^C-labeled cultures with high-resolution mass spectrometry. Anal Chem.

[52] Allen DK, Evans BS, Libourel IG (2014). Analysis of isotopic labeling in peptide fragments by tandem mass spectrometry. PLoS ONE.

[53] Hollinshead WD, Rodriguez S, Martin HG, Wang G, Baidoo EEK, Sale KL, Keasling JD, Mukhopadhyay A, Tang YJ (2016). Examining *Escherichia coli* glycolytic pathways, catabolite repression, and metabolite channeling using ∆*pfk* mutants. Biotechnol Biofuels.

[54] Bennett BD, Yuan J, Kimball EH, Rabinowitz JD (2008). Absolute quantitation of intracellular metabolite concentrations by an isotope ratio-based approach. Nat Protoc.

[55] Wu L, Mashego MR, van Dam JC, Proell AM, Vinke JL, Ras C, van Winden WA, van Gulik WM, Heijnen JJ (2005). Quantitative analysis of the microbial metabolome by isotope dilution mass spectrometry using uniformly ^13^C-labeled cell extracts as internal standards. Anal Biochem.

[56] You L, Page L, Feng X, Berla B, Pakrasi HB, Tang YJ. Metabolic pathway confirmation and discovery through ^13^C-labeling of proteinogenic amino acids. J Vis Exp. 2012;59:e3583. 10.3791/3583 PMC3462576 22314852

