# Supplementary material for: Deciphering cyanobacterial phenotypes for fast photoautotrophic growth via isotopically nonstationary metabolic flux analysis
Source: Biotechnol Biofuels. 2017 Nov 16;10:273. doi: 10.1186/s13068-017-0958-y (PMC5691832; doi:10.1186/s13068-017-0958-y)
Supplement: Supplementary file 1 — Additional file 1. Deciphering cyanobacterial phenotypes for fast photoautotrophic growth via isotopically nonstationary metabolic flux analysis: Supporting Information. Supporting information contains biomass measurements, model development, Supporting Tables 1–5, Supporting Figures 1–7, and a Supporting photo. [file 13068_2017_958_MOESM1_ESM.docx]

**Deciphering cyanobacterial phenotypes for fast photoautotrophic growth via isotopically nonstationary metabolic flux analysis**

Mary H. Abernathy, Jingjie Yu, Fangfang Ma, Michelle Liberton, Justin Ungerer,

Whitney Hollinshead, Saratram Gopalakrishnan, Lian He, Costas D. Maranas,

Himadri B. Pakrasi, Doug K. Allen, and Yinjie J. Tang

**Part 1: Supporting Information for Measurement and Modeling Methods**

**Biomass composition analysis.** Protein and amino acid compositional analysis was performed by the Molecular Structure Facility, University of California (Davis, CA) and measured by hydrolysis and oxidation with performic acid. For lipid measurement, biomass was mixed with chloroform-methanol-water (2:1:0.8) and shaken (200 rpm) for 3 h. After the complete extraction of lipids from the biomass, the resulting chloroform layer was dried to determine the weight of the lipid residue. The fatty acid composition was then analyzed via GC-MS after transesterification. Glycogen was analyzed colorimetrically at 680 nm as a reducing sugar based on a previously published anthrone protocol (1). Chlorophyll *a* was extracted with 100% methanol in the dark for 24 h. The concentration was determined spectrophotometrically at 665 and 652 nm according to previous literature (2). Other photosynthetic pigments were not accounted in the lumped biomass equation due to minimal biomass contribution (3). Ash weight of the biomass samples was calculated from the mass difference after complete combustion. Total carbohydrate was measured based on sulfuric acid hydrolysis protocols (www.nrel.gov/biomass/analytical_procedures.html), which was performed by Dr. Wei Liao group at Michigan State University. Nucleotide contents were estimated based on mass balance (4).

**Ion-pairing LC-MS/MS method:** For LC-MS analysis, the pelleted samples were extracted with a chloroform-methanol method (5). The aqueous phase from the extraction was transferred into GC vials, lyophilized, and stored at -80 ^o^C. Dried samples were reconstituted in water for LC-MS/MS analysis. Ion-pairing LC-MS/MS was performed on a Shimadzu HPLC linked to an ion-trap triple quadruple MS/MS system (4000 QTRAP and 6500 QTRAP, AB Sciex Instruments) at the Proteomics and Mass Spectrometry Facility, Donald Danforth Plant Science Center, St. Louis, MO. The mobile phases utilized for a gradient elution were 10 mM tributylamine (Acros, Belgium) and 11 mM acetic acid (A) and methanol (B). A Synergi Hydro-RP column (80 Å, 150 x 2.00 mm, Phenomenex Inc., CA) was used with the following gradient profile: 0% B (0-5 min), 0-45% B (5-50 min), 45-90% B (50-52 min), 90% B (52-57 min), 90-0% B (57-59 min), 0% B (59-64 min). Negative ionization mode and multiple-reaction monitoring mode were used, and all data acquisition and analysis was performed on the Analyst software (AB/MDS Sciex).

**Mutant Construction.** For the *zwf* mutant, a plasmid was designed to target a 1000 bp region (extending from nucleotides 41-1041) of the *zwf* open reading frame for replacement with a kanamycin resistance cassette by double homologous recombination. A circular polymerase extension cloning (CPEC) method (6) was used to generate an upstream (1000 bp)-kanamycin-downstream (1000 bp) construct in pBR322. Sequencing and restriction digestion were used to confirm the resulting plasmid (pSL2480), which was introduced into *Synechococcus* 2973 cells by tri-parental conjugation as previously described (7). To create the cargo strain, the pSL2480 plasmid was first transformed into competent HB101 *E. coli* cells containing the pRL623 helper plasmid. Overnight cultures (100 µL each) of the cargo strain and the conjugal strain (pRL443) were washed with distilled water and mixed with 200 μl of washed *Synechococcus* 2973 cells. Cell mixtures were plated on BG-11 + 5% LB (v/v) agar plates containing 10 µg/ml kanamycin. Plates were placed under continuous illumination (100 μmol photons•m^-2^•s^-1^) at 38 °C. Mutant colonies appeared within 4-5 days and segregation was confirmed by PCR.

A *cpf1* based CRISPR system was used to delete 6-phosphogluconolactonase and succinate dehydrogenase from start codon to stop codon from the chromosome (8). In brief, oligos targeting the gene’s coding region were cloned into the aarI sites on pSL2680 to target the nuclease to the chromosomal region containing the gene of interest. Next a region 2kb in length containing 1kb of upstream and downstream sequences were assembled into the resulting plasmid using Gibson assembly. The resulting plasmid was introduced into *Synechococcus* 2973 cells by tri-parental conjugation. After repatchings onto selective media, patches were sequenced and a patch that was positive for the deletion was started in BG-11 without antibiotics and grown to an OD_730_ of 1.0. Serial dilutions were plated to obtain single colonies. Colonies that were cured of the editing plasmids were then sequenced to verify the deletion of 6-phosphogluconolactonase and succinate dehydrogenase.

**INST-MFA model formulation:** The INCA model listed in Supporting Table 1 and its results listed in Supporting Table 3, 4 and 5, along with the lumped biomass equation generated from Supporting Table 2 are based on the following:

1. The INCA platform is convenient because it does not need to estimate metabolite pool sizes; however, the platform has to solve numerically stiff ODE equations for isotopomer changes through very different time scales of metabolite labeling (9). Therefore, it is computationally expensive for flux calculations and confidence interval estimations.
2. In the culture medium, it is assumed there was residual ^12^CO_2_ after the pulse of saturated ^13^C-bicarbonate. The INCA model was allowed to vary the unlabeled carbon input between 0-35% based on steady-state labeling. The best fit values across all models were obtained when ^12^CO_2_ input was 3% of total carbon fixation. This result is consistent to the fact that labeled bicarbonate concentration was much higher than dissolved ^12^CO_2_ in the culture.
3. Metabolite levels and fluxes were assumed to be constant throughout the experiment and were not perturbed by the addition of sodium bicarbonate. The effect of isotope discrimination was assumed to be negligible.
4. Succinate and fumarate are symmetrical molecules, and this was accounted for within the INCA model.
5. It is unknown whether the TCA cycle in *Synechococcus* is closed by the GABA shunt (10) or the oxoglutarate dehydrogenase pathway (11); thus the reaction network was modeled as AKG 🡪 SUC + CO_2_, lumping both shunts into one closed TCA reaction. The SSR of the model was not changed when this reaction was removed, obtaining a p-value of 6.5•10^-1^ based on the chi-square statistical test with one degree of freedom. This indicates that the flux was not significantly different from zero. However, the sequential labeling of free glutamate and succinate suggests a low, yet measurable GABA shunt in *Synechococcus* 2973 (Supporting Figure 4).
6. Although there is limited to no evidence for a bacterial glyoxylate shunt in *Synechococcus*, we included this pathway in the model to be consistent with the previous work. Addition of the glyoxlate shunt only slightly changes model fitting quality, while the resulting flux of glyoxylate shunt was still close to zero (*p* < 0.001, based on chi-square statistical test with two DOFs).
7. To test the presence of OPPP, we removed these two equations (G6P🡪6PG, 6PG🡪Ru5P +CO2) from the model. This resulted in reduced set of DOFs. Without the OPPP, the model fitting quality was not significantly changed. This indicates very little flux through OPPP and was not a consequence of the choice of measurements because oxidation that produces CO_2_ and a pentose phosphate would be unique to the labeling in the Calvin cycle. This was additionally confirmed by OPPP knock out mutant, Δ*zwf*.
8. Confidence intervals were evaluated using two different methods due to numerical stiffness problems that were encountered. The approaches gave similar results and therefore served to confirm each other. Numerical stiffness problems are expected due to label accumulation processes occurring over a large range of time scales. To solve the entire non-stationary isotopomer reaction network with high numerical precision is much more difficult than solving for steady state isotopomer balance equations (9). Thus, in addition to parameter continuation, we used the Monte Carlo method to examine to confidence intervals based on potential measurement timing errors (12). Specifically, the sample quenching time points were perturbed by normally distributing noise within measurement timing variations (±3 seconds). INCA was then restarted for flux calculations. By repeatedly running INCA using perturbed timing of isotopomer data, the resulting distribution of flux values allowed for the estimation of flux standard errors when measuring the rapid turnover of intracellular metabolites subjected to manual errors as seen in Supporting Table 3. Additionally, while INCA-based MFA did not require the pool size estimation, if we added estimated pool sizes (MAL, PEP, AcCoA, SUC, CIT) from isotopomer ratio analysis (13), the flux results from PBRs had improved SSR and facilitated determination of unresolved confidence intervals (Supporting Table 3). As expected, net fluxes remained relatively similar to flux results without pool size constraints.
9. The uneven distribution of the residuals in the shaking flask model demonstrated that they were less consistent and of reduced quality (Supporting Figure 3). Dilution factors (14) were required for several metabolites that were significantly less labeled than their downstream products, including: 3PGA, F6P, FBP, and SBP (Figure 5). In INCA platform, dilution parameters are often required to describe the lack of equilibrium between labeled and unlabeled pools or labeling dilution from unknown resources. The dilution factors were more crucial for the shaking flask conditions, which represented a higher degree of inactive metabolite pools. Besides the inactive pools prescribed for the PBR, an additional dilution for DHAP, CIT, PEP, and MAL improved the SF-based model fitting. Poor quality of SF flux results is not surprising because sub-optimal and light-limiting SF conditions causes sub-populations of cyanobacteria with different metabolisms (15).
10. Several assumptions were made when quantifying the biomass components. Glycogen was assumed to have a molecular weight of 666.58 g/mol, chlorophyll a was assumed to have a molecular weight of 893.5 g/mol, lipids were assumed to have a molecular weight of 270 g/mol, DNA was assumed to have a molecular weight of 487 g/mol, and RNA was assumed to have a molecular weight of 499.5 g/mol.

**Biomass Composition Analyses of *Synechococcus* 2973 and *Synechococcus* 7942.** Standard deviations are reported from biological duplicates to quadruplicates. ^†^ or ^*^ indicates p-value <0.05 using two-tailed equal variance Student’s t-Test.

| **Biomass components (unit: % of gDCW)** | | ***Syn. 2973-* Photobioreactor** | ***Syn. 2973-* Shaking Flask** | ***Syn. 7942-* Photobioreactor** |  |
| --- | --- | --- | --- | --- | --- |
| Protein | | 52.7 ± 2.5 | 49.4 ± 2.1 | 40.9 ± 0.4 |  |
| Lipid | | 9.3 ± 0.6 | 13.2 ± 2.4 | 10.7 ± 0.6 |  |
| Glycogen | | 1.5 ± 0.5^†*^ | 6.0 ± 1.0^*^ | 12.6 ± 4.3^†^ |  |
| Other sugars | | 6.5 ± 0.7 | NA | NA |  |
| Ash | | 7 ± 1 | NA | NA |  |
| Chlorophyll *a* % | | 1.2 ± 0.2 | 1.0 ± 0.3 | 1.2 ± 0.1 |  |
| Chlorophyll *a* (μg/mL/OD_730_) | | 4.4 ± 0.5^*^ | 7.4 ± 0.3^*^ | 5.2 ± 0.6 |  |
| RNA/DNA | | 21.8^a^ | 16.9^a^ | 21.1^a^ |  |
| **Amino Acid profiles (unit: mmol/gDCW)** | | ***Syn. 2973-* Photobioreactor** | ***Syn. 2973-* Shaking Flask** | ***Syn. 7942-* Photobioreactor** |  |
| Glycine | | 0.44 ± 0.02 | 0.48 ± 0.07 | 0.34 ± 0.00 |  |
| Proline | | 0.21 ± 0.01 | 0.24 ± 0.04 | 0.16 ± 0.00 |  |
| Alanine | | 0.62 ± 0.03 | 0.67 ± 0.08 | 0.49 ± 0.01 |  |
| Valine | | 0.31 ± 0.01 | 0.35 ± 0.06 | 0.25 ± 0.01 |  |
| Leucine | | 0.46 ± 0.02 | 0.53 ± 0.08 | 0.37 ± 0.01 |  |
| Isoleucine | | 0.23 ± 0.01 | 0.27 ± 0.04 | 0.19 ± 0.01 |  |
| Methionine | | 0.08 ± 0.00 | 0.08 ± 0.01 | 0.06 ± 0.01 |  |
| Cysteine | | 0.04 ± 0.00 | 0.04 ± 0.01 | 0.03 ± 0.00 |  |
| Phenylalanine | | 0.20 ± 0.01 | 0.24 ± 0.04 | 0.15 ± 0.00 |  |
| Tyrosine | | 0.16 ± 0.00 | 0.18 ± 0.03 | 0.13 ± 0.00 |  |
| Histidine | | 0.06 ± 0.00 | 0.08 ± 0.011 | 0.04 ± 0.00 |  |
| Lysine | | 0.21 ± 0.01 | 0.22 ± 0.04 | 0.16 ± 0.00 |  |
| Arginine | | 0.27 ± 0.01 | 0.28 ± 0.04 | 0.21 ± 0.00 |  |
| Glutamate and Glutamine | | 0.60 ± 0.05^*^ | 0.44 ± 0.05 | 0.36 ± 0.00^*^ |  |
| Aspartate and Asparagine | | 0.45 ± 0.02 | 0.49 ± 0.07 | 0.41 ± 0.01 |  |
| Serine | | 0.27 ± 0.02 | 0.30 ± 0.04 | 0.21 ± 0.00 |  |
| Threonine | | 0.29 ± 0.01 | 0.32 ± 0.05 | 0.22 ± 0.00 |  |
| **Fatty acid profiles (wt%)** | | ***Syn. 2973-* Photobioreactor** | ***Syn. 2973-* Shaking Flask** | ***Syn. 7942-* Photobioreactor** |  |
| C14:1 | 0.3 ± 0.1 | | 1.1 ± 0.4 | 2.5 ± 0.1 | |
| C14:0 | 1.2 ± 0.5 | | 0.7 ± 0.07 | 2.5 ± 0.3 | |
| C16:1 | 32.3 ± 0.1 | | 39.9 ± 1.4 | 35.0 ± 0.1 | |
| C16:0 | 53.3 ± 1.3 | | 56. 3 ± 0.6 | 51.4 ± 0.1 | |
| C18:1 | 9.4 ± 0.1 | | 1.8 ± 0.7 | 4.8 ± 0.4 | |
| C18:0 | 2.9 ± 0.9 | | 0.3 ± 0.3 | 3.1 ± 0.1 | |

NA: measured values from the biomass analysis service center had high noises.

^a^Estimated based on biomass composition and the reference (4). Previous study indicates that flux result is not sensitive to the measured nucleotide composition of the cells (16).

**Part 2: Supporting Figures and Tables**

**Supporting Table 1. A complete list of reactions and atom transitions for the *Synechococcus elongatus* UTEX 2973 metabolic network.**

**Glycolysis and the Oxidative Pentose Phosphate pathway**

| G6P (abcdef) | 🡨🡪 | F6P(abcdef) |
| --- | --- | --- |
| G6P (abcdef) | 🡪 | 6PG (abcdef) |
| 6PG (abcdef) | 🡪 | Ru5P (bcdef) + CO2 (a) |
| F6P (abcdef) | 🡨🡪 | FBP (abcdef) |
| FBP (abcdef) | 🡨🡪 | DHAP (cba) + GAP (def) |
| DHAP (abc) | 🡨🡪 | GAP (abc) |
| GAP (abc) | 🡨🡪 | 3PGA (abc) |
| 3PGA(abc) | 🡨🡪 | 2PGA (abc) |
| 2PGA (abc) | 🡨🡪 | PEP (abc) |
| PEP (abc) | 🡨🡪 | PYR (abc) |

**The Citric Acid Cycle and Amphibolic reactions**

| PYR (abc) | 🡪 | AcCoA(bc) + CO2 (a) |
| --- | --- | --- |
| OAA (abcd) + AcCoA (ef) | 🡪 | CIT (dcbfea) |
| CIT (abcdef) | 🡨🡪 | ICIT (abcdef) |
| ICIT (abcdef) | 🡪 | AKG (abcde) + CO2 (f) |
| AKG (abcde) | 🡪 | SUC (bcde) + CO2 (a) |
| SUC (abcd) | 🡨🡪 | FUM (abcd) |
| FUM (abcd) | 🡨🡪 | MAL (abcd) |
| MAL (abcd) | 🡨🡪 | OAA (abcd) |
| MAL (abcd) | 🡪 | PYR (abc) + CO2 (d) |
| PEP (abc) + CO2 (d) | 🡨🡪 | OAA (abcd) |
| ICIT (abcdef) | 🡪 | SUC (dcef) + GLX(ab) |
| GLX (ab) + ACA (cd) | 🡪 | MAL (abdc) |

**Calvin-Benson-Bassham Cycle**

| Ru5P (abcde) | 🡨🡪 | X5P(abcde) |
| --- | --- | --- |
| Ru5P (abcde) | 🡨🡪 | R5P (abcde) |
| Ru5P (abcde) | 🡪 | RuBP (abcde) |
| RuBP (abcde) + CO2 (f) | 🡪 | 3PGA (cde) +3PGA (fba) |
| X5P (abcde) | 🡨🡪 | GAP (cde) + EC2 (ab) |
| F6P (abcdef) | 🡨🡪 | E4P (cdef) + EC2 (ab) |
| S7P (abcdefg) | 🡨🡪 | R5P (cdefg) + EC2(ab) |
| F6P (abcdef) | 🡨🡪 | GAP (def) + EC3 (abc) |
| S7P (abcdefg) | 🡨🡪 | E4P (defg) +EC3 (abc) |
| DHAP (abc) + E4P (defg) | 🡪 | SBP (cbadefg) |
| SBP (abcdefg) | 🡪 | S7P (abcdefg) |

**Photorespiration**

| RuBP (abcde) | 🡪 | 3PGA(cde) + 2PG (ba) |
| --- | --- | --- |
| 2PG (ab) | 🡪 | GLC (ab) |
| GLC (ab) | 🡪 | GLX (ab) |
| GLX (ab) + GLX (cd) | 🡪 | GA (abd) + CO2 (c)0. |
| GA (abc) | 🡨🡪 | 2PGA (abc) |

**Glycogen Synthesis and breakdown**

| G6P (abcdef) | 🡨🡪 | G1P(abcdef) |
| --- | --- | --- |
| G1P (abcdef) | 🡨🡪 | GLYC (abcdef) |

**Biosynthesis *Syn*. 2973-Photobioreactor**

| 0.404*R5P + 3.132*AcCoA + 0.12*G6P + 0.358*E4P + 0.519*3PGA + 0.715*PEP + 3.234*PYR + 1.477*OAA + 0.760*αKG + 0.080*GAP | 🡪 | Biomass + 0.522*FUM + 1.850*CO_2_ |
| --- | --- | --- |

**Biosynthesis *Syn*. 7942-Photobioreactor**

| 0.465*R5P + 3.789*AcCoA + 0.286*G6P + 0.275*E4P + 0.461*3PGA + 0.550*PEP + 2.698*PYR + 1.154*OAA + 0.805*αKG + 0.218*GAP | 🡪 | Biomass + 0.528*FUM + 1.369*CO_2_ |
| --- | --- | --- |

**Biosynthesis *Syn*. 2973-Shaking Flask**

| 0.426*R5P + 4.846*AcCoA + 0.188*G6P + 0.417*E4P + 0.580*3PGA + 0.834*PEP + 3.701*PYR + 1.313*OAA + 0.868*αKG + 0.175*GAP | 🡪 | Biomass + 0.536*FUM + 2.142*CO_2_ |
| --- | --- | --- |

**Supporting Table 2. Equations used to form the lumped biomass equation.** The equations represent the formation of amino acids, nucleotides, photosynthetic pigments, and lipids to central metabolites. Using measured and literature values for amino acids, carbohydrates, lipids, nucleotides and pigments, the stoichiometric molar quantity for central metabolites per 1 kg of DCW was determined.

| Amino Acids (4) | Carbohydrates and lipids (4) | Nucleotides (17)  and pigments |
| --- | --- | --- |
| GLU + ATP + 2*NADPH => PRO + NADP^+^ + ADP | G6P => Carbohydrate | R5P + 1.2*ASN + 0.25*SER + 2*GLN + 0.25*GLY + FTHF  + 0.5*CO_2_ + 2.3*H_2_O + NADPH + 0.76*NAD^+^ + 8*ATP => DNA + 0.75*FUM + 2*GLU + THF  + 8*ADP + 0.76*NADH + NADP^+^ |
| PYR + GLU => ALA + AKG | 7*AcCoA + 12*NADPH  =>C14 +7*CoA + 12*NADP^+^ | R5P + 1.2*ASN + 2.1*GLN + 0.54*GLY + 1.1*FTHF  + 0.54*CO_2_ + 2.2*H_2_O + 0.79*NAD^+^ + 8.2*ATP => RNA + 0.75*FUM + 2.1*GLU + 1.1*THF + 8.2*ADP + 0.79*NADH |
| 2*PYR + GLU => VAL + CO_2_ + AKG | 7*AcCoA+12*NADPH  => C14 + 7*CoA + 12*NADP^+^ | 8Glu+4GAP+4PYR=>Chl a |
| 2*PYR + AcCoA + GLU => 2*CO_2_ + AKG + LEU + CoA | 8*AcCoA+14*NADPH  => C16 + 8*CoA + 14*NADP^+^ |  |
| THR + NH_3_ + PYR + GLU + 6*NADPH => ILE + AKG + CO_2_ | 8*AcCoA + 14*NADPH  => C16 + 8*CoA + 14*NADP^+^ |  |
| ASP + SUCCoA + CYS + 5*MeTHF + ATP + 2*NADPH => MET + SUC + CoA + PYR + NH_3_ + THF + ADP + 2*NADP^+^ | 8.5*AcCoA + 15*NADPH => C17 + 8.5*CoA+ 15*NADP^+^ |  |
| CHM + GLU => PHE + AKG + CO_2_ | 8.5*AcCoA + 15*NADPH => C17 + 8.5*CoA + 15*NADP^+^ |  |
| CHM + GLU + NAD^+^ => TYR + AKG + CO_2_ + NADH | 9*AcCoA + 16*NADPH  => C18 + 9*CoA + 16*NADP^+^ |  |
| R5P + GLN + 2*ATP + 2*NAD^+^ => HIS + GLU + 2*PP + AICAR + 2*NADH + 2*ADP | 9*AcCoA + 16*NADPH  => C18 + 9*CoA + 16*NADP^+^ |  |
| ASP + SUCCoA + GLU + PYR + 2*NADPH + ATP => LYS + AKG + CoA + CO_2_ + SUC |  |  |
| 2*GLU + AcCoA + CP + ASP + 2*ATP + NADPH => ARG + AKG + FUM + Ac + CoA + 2*ADP + NADP^+^ |  |  |
| SER => GLY |  |  |
| AKG + 2*NH_3_ + NADPH + ATP => GLN + NADP^+^ + ADP |  |  |
| OAA + GLU => ASP+AKG |  |  |
| NH_3_ + ASP + ATP => ASN + ADP |  |  |
| SER + FTHF + GLY => MeTHFA |  |  |
| E4P + 2*PEP + ATP + NADPH => CHM + ADP |  |  |
| 3PGA + GLU + NAD^+^ => SER + AKG + NADH |  |  |
| SER + AcCoA => Cys + Ac + CoA |  |  |
| ASP + 2*ATP + 2*NADPH => THR |  |  |

**Supporting Table 3. Net fluxes and dilution parameters determined by ^13^C INST-MFA for the *Synechococcus* 2973 photobioreactor model.** Relative mean values are net fluxes relative to a net CO_2_ uptake rate of 100 (actual uptake rate was estimated as 12.2 mmol/g-DW/h and those mean values are reported as well). Mean parameter estimates and 95% confidence bounds using INCA’s parameter continuation method are shown below. Dilution parameters represent the fraction of active pool in the cell. Although INCA platform does not need pool size measurement, including the pool size data can improve the model confidence interval. For example, * indicates a net flux where the 95% confidence intervals were not resolved. Constraining several pool size measurements with estimated values (MAL, PEP, AcCoA, SUC, CIT) resulted in the best fit model and resolved the confidence interval issue and are noted by *. In addition, average flux values and their flux ranges were also calculated via Monte Carlo method as stated above (randomly perturbed sampling time within measurement errors, n=50). The Monte Carlo based flux estimation gives the possible flux ranges due to imprecise timing during sample harvesting processes. In the Figure 2 of the main text, we reported the flux results without constraining the pool sizes.

| **Reaction** | **Relative mean values** | | **Confidence Intervals 95%** | ***Monte Carlo based flux estimation*** | **Absolute mean values** |
| --- | --- | --- | --- | --- | --- |
|  | **Pool size**  **not**  **constrained** | **Pool size**  **constrained** |  |  |  |
| G6P <-> G1P | 0.0 | 0.0 | 0.9, 0.0 | ***0.0 ± 0.1*** | 0.0 |
| G1P <-> GLYC | 0.0 | 0.0 | 0.9, 0.0 | ***0.0 ± 0.1*** | 0.0 |
| G6P <-> F6P | -0.1 | -0.1 | -0.9, -0.1 | ***-0.1 ± 0.06*** | -0.01 |
| G6P -> 6PG | 0.0 | 0.0 | 0.0, 0.09 | ***0.0 ± 0.06*** | 0.0 |
| 6PG -> RU5P + CO2 | 0.0 | 0.0 | 0.0, 0.09 | ***0.0 ± 0.06*** | 0.0 |
| F6P <-> FBP | -39.9 | -39.4 | -42.6, -34.5 | ***-41.0 ± 0.8*** | -5.1 |
| FBP <-> DHAP + GAP | -39.9 | -39.4 | -42.6, -34.5 | ***-41.0 ± 0.8*** | -5.1 |
| DHAP <-> GAP | -78.6 | -77.7 | -80.3, -75.3 | ***-79.6 ± 2.9*** | -10.0 |
| GAP <-> 3PGA | -197.4 | -195.2 | *-197.3, -188.8 | ***-199.9 ± 7.2*** | -25.1 |
| 3PGA <-> 2PGA | 25.0 | 25.0 | 23.7, 26.6 | ***25.0 ± 2.3*** | 3.4 |
| 2PGA <-> PEP | 29.3 | 28.8 | 28.9, 29.6 | ***29.7 ± 1.3*** | 3.8 |
| PEP <-> PYR | 22.0 | 19.7 | 21.0, 22.2 | ***20.9 ± 0.8*** | 2.6 |
| RU5P <-> X5P | -78.5 | -77.6 | -81.1, -76.4 | ***-79.5 ± 2.9*** | -10.0 |
| RU5P <-> R5P | -37.5 | -37.1 | -38.8, -36.5 | ***-37.8 ± 1.6*** | -4.8 |
| RU5P -> RUBP | 116.0 | 114.7 | 112, 119.6 | ***117.3 ± 4.4*** | 14.7 |
| RUBP + CO2 -> 3PGA + 3PGA | 107.9 | 107.1 | *105.5, 109.2 | ***110.3 ± 2.9*** | 13.9 |
| X5P <-> GAP + EC2 | -78.5 | -77.6 | -81.1, -76.4 | ***-79.5 ± 2.9*** | -10.0 |
| F6P <-> E4P + EC2 | 39.8 | 39.4 | 38.7, 41.1 | ***40.3 ± 1.4*** | 5.1 |
| S7P <-> R5P + EC2 | 38.7 | 38.3 | 37.7, 40.0 | ***39.2 ± 1.43*** | 4.9 |
| F6P <-> GAP + EC3 | 0.0 | 0.0 | 0.0, 2.7 | ***0.0 ± 0.8*** | 0.0 |
| S7P <-> E4P + EC3 | 0.0 | 0.0 | -2.7, 0.0 | ***0.0 ± 0.8*** | 0.0 |
| DHAP + E4P -> SBP | 38.7 | 38.3 | *35.3, 43.6 | ***39.5 ± 1.2*** | 4.9 |
| SBP -> S7P | 38.7 | 38.3 | *35.3, 43.6 | ***39.5 ± 1.2*** | 4.9 |
| PYR -> AcCoA + CO2 | 12.1 | 11.7 | 11.7, 12.7 | ***12.2 ± 0.8*** | 1.6 |
| OAA + AcCoA -> CIT | 2.7 | 2.3 | 2.3, 3.1 | ***2.6 ± 0.5*** | 0.4 |
| CIT <-> ICI | 2.7 | 2.3 | 2.3, 3.1 | ***2.6 ± 0.5*** | 0.4 |
| ICIT <-> AKG + CO2 | 2.3 | 2.3 | 2.3, 2.8 | ***2.3 ± 0.1*** | 0.3 |
| AKG -> SUC + CO2 | 0.0 | 0.0 | 0.0, 0.6 | ***0.0 ± 0.0*** | 0.0 |
| SUC <-> FUM | 0.4 | 0.0 | 0.0, 0.8 | ***0.3 ± 0.5*** | 0.1 |
| FUM <-> MAL | 2.0 | 1.6 | 1.6, 2.3 | ***1.9 ± 0.5*** | 0.3 |
| MAL <-> OAA | 2.0 | -0.3 | 1.3, 2.4 | ***0.5 ± 0.9*** | 0.0 |
| MAL -> PYR + CO2 | 0.0 | 1.9 | *0.0, 5.0 | ***1.4 ± 1.2*** | 0.3 |
| PEP + CO2 -> OAA | 5.1 | 7.0 | *5.1, 9.9 | ***6.7 ± 1.3*** | 1.0 |
| ICIT -> GLX + SUC | 0.4 | 0.0 | 0.0, 0.9 | ***0.3 ± 0.5*** | 0.1 |
| GLX + AcCoA -> MAL | 0.0 | 0.0 | *0.0, 0.3 | ***0.0 ± 0.0*** | 0.0 |
| RUBP -> 3PGA + 2PG | 8.1 | 7.6 | 6.8, 10.6 | ***9.1 ± 2.6*** | 0.8 |
| 2PG -> GLC | 8.1 | 7.6 | 6.8, 10.6 | ***9.1 ± 2.6*** | 0.8 |
| GLC -> GLX | 8.1 | 7.6 | 6.8, 10.6 | ***9.1 ± 2.6*** | 0.8 |
| GLX + GLX -> GA + CO2 | 4.2 | 3.8 | 3.1, 5.6 | ***4.7 ± 1.1*** | 0.5 |
| GA <-> 2PGA | 4.2 | 3.8 | 3.1, 5.6 | ***4.7 ± 1.1*** | 0.5 |
| 0.404*R5P + 3.132*AcCoA + 0.023*G6P + 0.358*E4P + 0.519*3PGA + 0.715*PEP + 3.234*PYR + 1.477*OAA + 0.760*AKG + 0.144*GAP -> Biomass + 0.522*FUM + 1.850*CO2 | 3.0 | 3.0 | 3.0, 3.0 | ***3.1 ± 0.1*** | 0.4 |
| *Dilution Parameters* |  |  |  |  |  |
| R5P | 0.72 | 0.72 | 0.68, 0.75 | ***0.78 ± 0.1*** | 0.75 |
| F6P | 0.78 | 0.78 | *0.74, 0.80 | ***0.79 ± 0.1*** | 0.78 |
| 3PGA | 0.73 | 0.73 | 0.69, 0.76 | ***0.78 ± 0.1*** | 0.79 |
| 2PG | 0.55 | 0.55 | 0.50, 0.60 | ***0.61 ± 0.2*** | 0.61 |
| SBP | 0.87 | 0.86 | 0.81, 0.91 | ***0.87 ± 0.3*** | 0.83 |
| FBP | 0.89 | 0.88 | 0.85, 0.94 | ***0.90 ± 0.1*** | 0.87 |

**Supporting Table 4. Net fluxes and dilution parameters determined by ^13^C INST-MFA for the *Synechococcus* 2973 shaking flasks.** Values are relative to a net CO_2_ uptake rate of 100 (actual uptake rate was estimated as 6.7 mmol/g-DW/h). Median parameter estimates and 95% confidence bounds using INCA’s parameter continuation method are shown. Due to suboptimal cultivation conditions, we did not perform further confidence interval analysis using fixed metabolite pool size or Monto Carlo methods.

| **Reaction** | **Mean value** | **LB 95%** | **UB 95%** |
| --- | --- | --- | --- |
| G6P <-> G1P | 0.0 | -1.25 | 0.23 |
| G1P <-> GLYC | 0.0 | -1.25 | 0.23 |
| G6P <-> F6P | -0.3 | -1.25 | 0.23 |
| G6P -> 6PG | 0.0 | 0.00 | 1.13 |
| 6PG -> RU5P + CO2 | 0.0 | 0 | 1.02 |
| F6P <-> FBP | -37.8 | NaN | -35.65 |
| FBP <-> DHAP + GAP | -37.8 | NaN | -35.65 |
| DHAP <-> GAP | -74.3 | -75.34 | -73.57 |
| GAP <-> 3PGA | -186.7 | -189.93 | -184.76 |
| 3PGA <-> 2PGA | 30.1 | 29.33 | NaN |
| 2PGA <-> PEP | 30.1 | 30.13 | NaN |
| PEP <-> PYR | 23.4 | 22.20 | 24.68 |
| RU5P <-> X5P | -74.1 | -75.59 | -73.34 |
| RU5P <-> R5P | -35.4 | -36.19 | -35.06 |
| RU5P -> RUBP | 109.5 | 107.99 | 11.52 |
| RUBP + CO2 -> 3PGA + 3PGA | 108.7 | 107.96 | NaN |
| X5P <-> GAP + EC2 | -74.1 | -75.59 | -73.34 |
| F6P <-> E4P + EC2 | 37.6 | 37.20 | 38.32 |
| S7P <-> R5P + EC2 | 36.7 | 36.14 | 37.27 |
| F6P <-> GAP + EC3 | 0.0 | -1.06 | 27.62 |
| S7P <-> E4P + EC3 | 0.0 | -27.62 | 1.06 |
| DHAP + E4P <-> SBP | 36.5 | 11.48 | 37.55 |
| SBP -> S7P | 36.5 | 11.48 | 37.55 |
| PYR -> AcCoA + CO2 | 15.3 | 14.93 | NaN |
| OAA + AcCoA -> CIT | 2.2 | 2.20 | NaN |
| CIT <-> ICIT | 2.2 | 2.20 | NaN |
| ICIT <-> AKG + CO2 | 2.2 | 2.20 | NaN |
| AKG -> SUC + CO2 | 0.0 | 0.00 | NaN |
| SUC <-> FUM | 0.0 | 0.00 | NaN |
| FUM <-> MAL | 1.4 | 1.37 | NaN |
| MAL <-> OAA | 1.0 | -0.23 | 2.18 |
| MAL -> PYR + CO2 | 1.2 | 0 | 2.84 |
| PEP + CO2 -> OAA | 4.6 | 3.40 | 6.95 |
| ICI -> GLX + SUC | 0.0 | 0 | 0.07 |
| GLX + AcCoA -> MAL | 0.8 | 0 | 1.36 |
| RUBP -> 3PGA + 2PG | 0.8 | NaN | 1.43 |
| 2PG -> GLC | 0.8 | NaN | 1.43 |
| GLC -> GLX | 0.8 | NaN | 1.43 |
| GLX + GLX -> GA + CO2 | 0.0 | 0.00 | 0.93 |
| GA <-> 2PGA | 0.0 | 0 | 0.93 |
| 0.426*R5P + 4.846*AcCoA + 0.090*G6P + 0.417*E4P + 0.580*3PGA + 0.834*PEP + 3.701*PYR + 1.313*OAA + 0.868*AKG + 0.175*GAP -> Biomass + 0.536*FUM + 2.142*CO2 | 2.5 | 2.53 | 2.53 |
| *Dilution Parameters* |  |  |  |
| R5P | 0.86 | 0.77 | 1.00 |
| F6P | 0.55 | 0.46 | 0.65 |
| 3PGA | 0.63 | 0.54 | NaN |
| DHAP | 0.21 | 0.10 | 0.31 |
| SBP | 0.46 | 0.37 | 0.53 |
| FBP | 0.52 | 0.42 | 0.62 |
| CIT | 0.12 | 0.00 | 1 |
| MAL | 0.66 | 0.00 | 1 |

Supporting Table 5. Net fluxes and dilution parameters determined by ^13^C INST-MFA for the *Synechococcus* 7942 photobioreactor model. Values are relative to a net CO_2_ uptake rate of 100 (actual uptake rate was estimated as 5.1 mmol/g-DW/h and those absolute mean values are reported as well). Median parameter estimates and 95% confidence bounds using INCA’s parameter continuation method are shown.

| **Reaction** | **Relative mean value** | **Confidence Intervals 95%** | **Absolute mean values** |
| --- | --- | --- | --- |
|  | **PS not**  **constrained** |  |  |
| G6P <-> G1P | 0.0 | -9.9, 0.0 | 0.0 |
| G1P <-> GLYC | 0.0 | -9.9, 0.0 | 0.0 |
| G6P <-> F6P | -0.7 | -9.9, -0.62 | -0.04 |
| G6P -> 6PG | 0.0 | 0.0, 9.3 | 0.005 |
| 6PG -> RU5P + CO2 | 0.0 | 0.0, 9.3 | 0.005 |
| F6P <-> FBP | -16.3 | -24.4, -16.2 | -2.0 |
| FBP <-> DHAP + GAP | -16.3 | -24.4, -16.2 | -2.0 |
| DHAP <-> GAP | -77.3 | NaN, -77.2 | -4.0 |
| GAP <-> 3PGA | -194.2 | NaN, -194.1 | -10.1 |
| 3PGA <-> 2PGA | 30.5 | 27.8, 32.3 | 1.6 |
| 2PGA <-> PEP | 30.5 | 30.5, 32.3 | 1.6 |
| PEP <-> PYR | 22.2 | 20.4, NaN | 1.2 |
| RU5P <-> X5P | -76.7 | NaN, -76.7 | -4.0 |
| RU5P <-> R5P | -36.4 | NaN, -36.4 | -1.9 |
| RU5P -> RUBP | 113.1 | 113.0, 123.5 | 5.9 |
| RUBP + CO2 -> 3PGA + 3PGA | 113.1 | 113.0, 123.5 | 5.9 |
| X5P <-> GAP + EC2 | -76.7 | NaN, -76.7 | -4.0 |
| F6P <-> E4P + EC2 | 38.8 | 38.8, NaN | 2.0 |
| S7P <-> R5P + EC2 | 37.9 | 37.9, NaN | 2.0 |
| F6P <-> GAP + EC3 | -23.1 | -38.9, NaN | -0.1 |
| S7P <-> E4P + EC3 | 23.1 | NaN, 38.9 | 0.1 |
| DHAP + E4P -> SBP | 61.0 | 15.5, 76.9 | 2.0 |
| SBP -> S7P | 61.0 | 15.5, 76.9 | 2.0 |
| PYR -> AcCoA + CO2 | 15.1 | 15.1, NaN | 0.8 |
| OAA + AcCoA -> CIT | 2.7 | 2.6, 4.5 | 0.2 |
| CIT <-> ICI | 2.7 | 2.6, 4.5 | 0.2 |
| ICIT <-> AKG + CO2 | 2.7 | 2.6, 4.5 | 0.2 |
| AKG -> SUC + CO2 | 0.0 | 0.0, 1.9 | 0.03 |
| SUC <-> FUM | 0.0 | 0.0, 1.9 | 0.03 |
| FUM <-> MAL | 1.7 | 1.7, 3.6 | 0.1 |
| MAL <-> OAA | 0.0 | -1.8, 1.2 | 0.1 |
| MAL -> PYR + CO2 | 1.7 | 0.0, 3.7 | 0.02 |
| PEP + CO2 -> OAA | 6.4 | 5.0, 8.4 | 0.3 |
| ICIT -> GLX + SUC | 0.0 | 0.0, 0.002 | 0.0 |
| GLX + AcCoA -> MAL | 0.0 | 0.0, 0.04 | 0.0 |
| RUBP -> 3PGA + 2PG | 0.0 | 0.0, 0.3 | 0.002 |
| 2PG -> GLC | 0.0 | 0.0, 0.3 | 0.002 |
| GLC -> GLX | 0.0 | 0.0, 0.3 | 0.002 |
| GLX + GLX -> GA + CO2 | 0.0 | 0.0, 5.0 | 0.001 |
| GA <-> 2PGA | 0.0 | 0.0, 5.0 | 0.001 |
| 0.465*R5P + 3.789*AcCoA + 0.188*G6P + 0.275*E4P + 0.461*3PGA + 0.550*PEP + 2.698*PYR + 1.154*OAA + 0.805*AKG + 0.218*GAP -> Biomass + 0.528*FUM + 1.369*CO2 | 3.3 | 3.3, 3.3 | 0.17 |
| *Dilution Parameters* |  |  |  |
| PEP | 0.77 | 0.71, 0.82 | 0.77 |
| RuBP | 0.64 | 0.57, 0.70 | 0.49 |
| 3PGA | 0.74 | 0.70, 0.79 | 0.72 |
| S7P | 0.57 | 0.51, 0.63 | 0.47 |
| SBP | 0.54 | 0.46, 0.58 | 0.48 |
| FBP | 0.51 | 0.44, 0.54 | 0.49 |

**Supporting Figure 1. Comparison of CO_2_ uptake rate measured by gas chromatography headspace/chlorophyll *a* content and estimated from the biomass composition.** Total CO_2_ was calculated from previously described methods as the sum of the dissolved and gaseous CO_2_ in a culture (18). Standard error bars were based on measured standard deviations from biomass composition of 5 biological replicates; *represents a p-value of <0.02 using two-tailed equal variance Student’s t-Test.


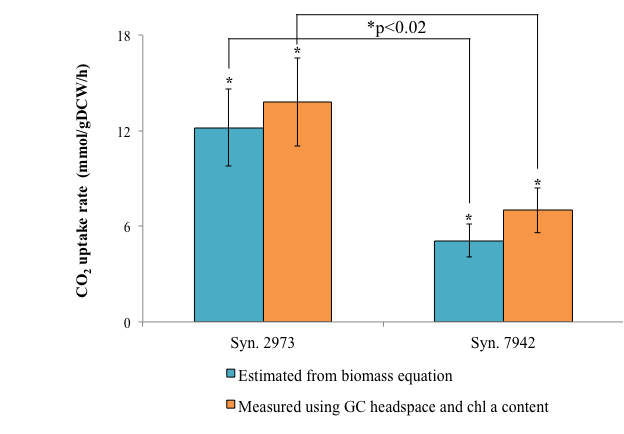


Supporting Figure 2. *Synechococcus* 2973 metabolites analyzed by LC-MS. A) FBP- the sample was extracted by methanol quenching method. 1 e4 represents its intensity (cps). B) FBP- the sample was extracted by liquid nitrogen quenching. 4.5 e4 represents its intensity (cps). C) PEP- metabolite pool size estimation by extracting a mixture of labeled *E. coli* and non-labeled *Synechococcus* 2973 cultures. (D-F) are chromatograms of 6PG (6PG standard, 6PG from *Synechococcus* 2973, and 6PG from *Synechococcus* 2973 Δ*zwf*).


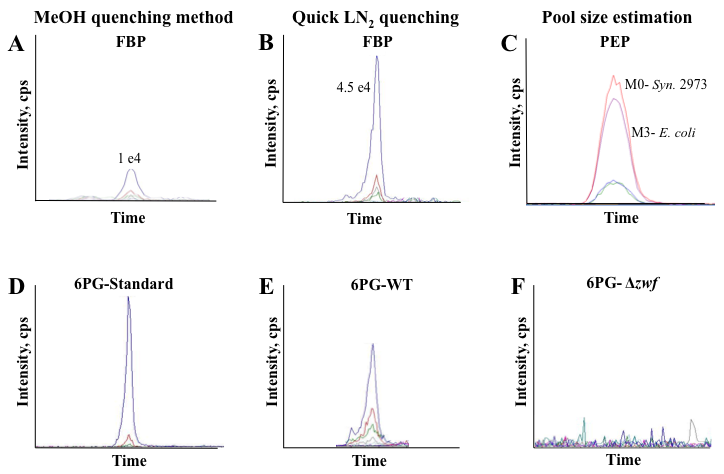


**Supporting Figure 3. A. The reported sum of square of residuals (SSR) for isotopomer fitting of individual metabolite from INCA modeling simulations (without adding the pool sizes as constraints).** The PBR had a smaller SSR (713), while the SF model had much higher SSR (981).


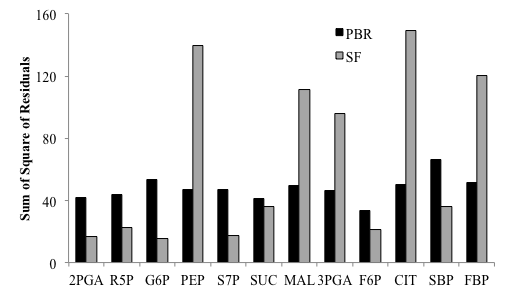


**Supporting Figure 4. Dynamic labeling patterns from INCA for all metabolites measured using IP-LC-MS/MS or HILIC-MS for *Synechococcus 2973* under PBR conditions.**  Experimentally measured MIDs with error bars representing standard measurement errors from biological duplicates. PGA (185), 6PG (275), PEP (167), G6P (259), R5P (229), S7P (289), SBP (269), SUC (118), MAL (134), F6P (259), CIT (192), FBP (339), G1P (259), 2PG (155), are all the metabolites listed with their nominal masses of M0 mass isotopomer shown in parentheses.


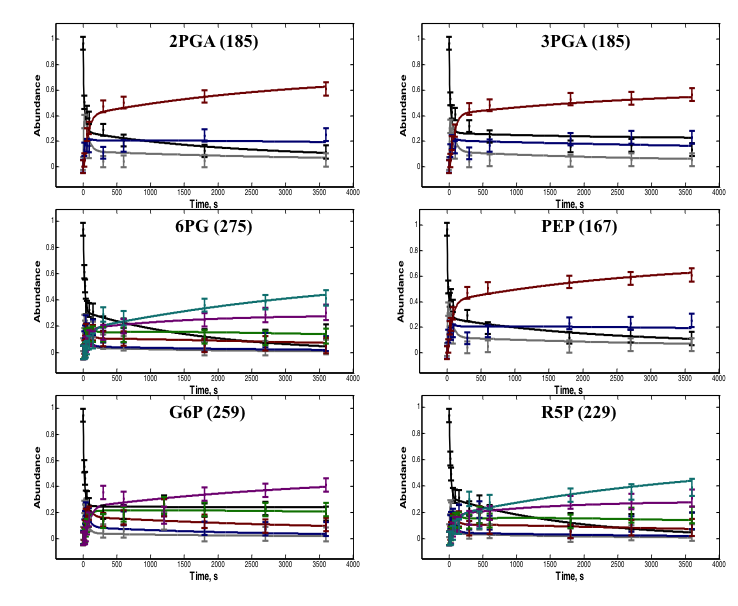

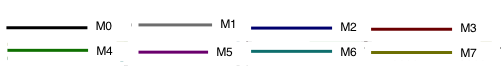


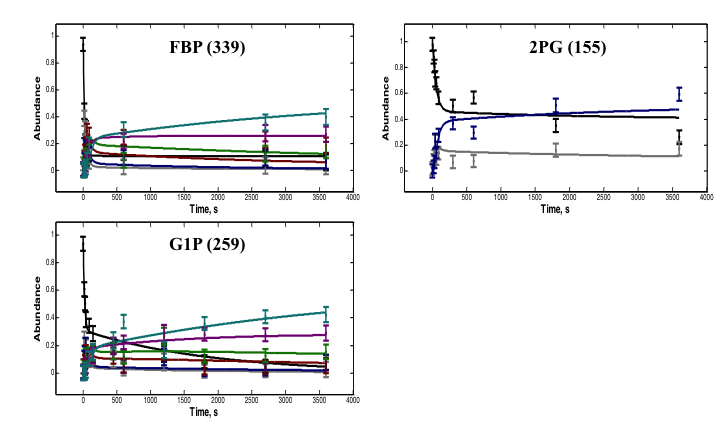

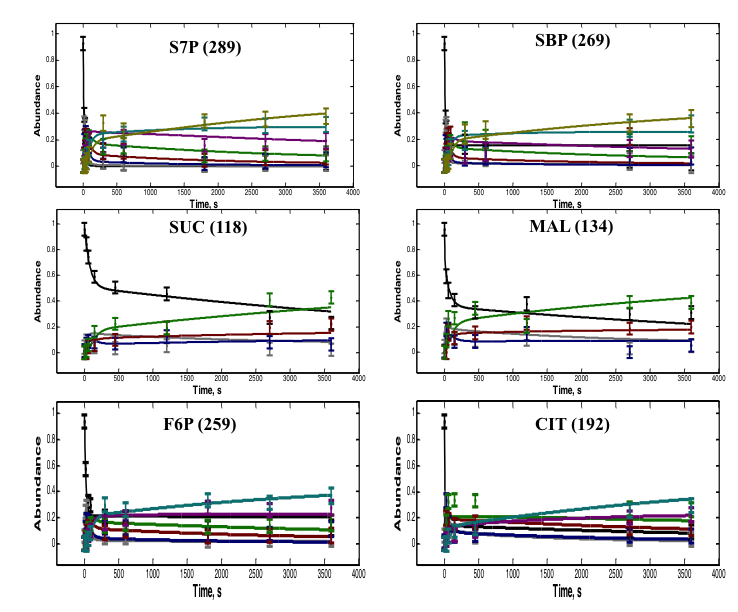


**Supporting Figure 5. The average percent ^13^C-enrichment of Citrate, Malate, Succinate, and free Glutamate in the photobioreactor conditions for *Synechococcus* 2973 and *Synechococcus* 7942 as a function of time.** Average ^13^C-Enrichment is calculated from experimentally measured MIDs where N is the number of carbons, M_i_ is the MID of the i*th* isotopomer, average ^13^C-Enrichment is $\frac{1}{N}\cdot\sum_{i=1}^{N} M_{i}\cdot i$ , and error bars represent average standard deviations from biological duplicates.


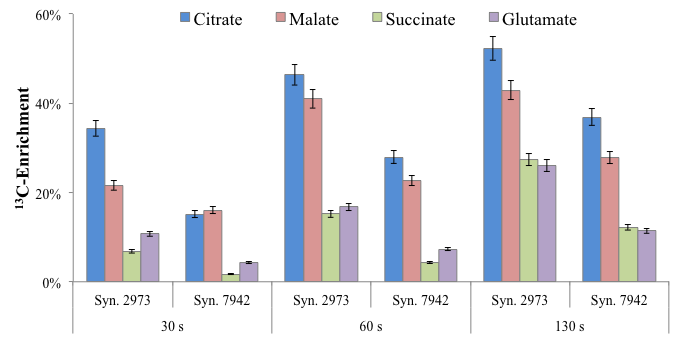


**Supporting Figure 6. Average ^13^C-enrichment of metabolite intermediates in the CBB cycle and PPP, in *Synechococcus* 2973 and *Synechococcus* 7942.** Average ^13^C-Enrichment is calculated from experimentally measured MIDs where N is the number of carbons, M_i_ is the MID of the i*th* isotopomer, average ^13^C-Enrichment is $\frac{1}{N}\cdot\sum_{i=1}^{N} M_{i}\cdot i$ , and error bars represent average standard deviations from biological duplicates.

**
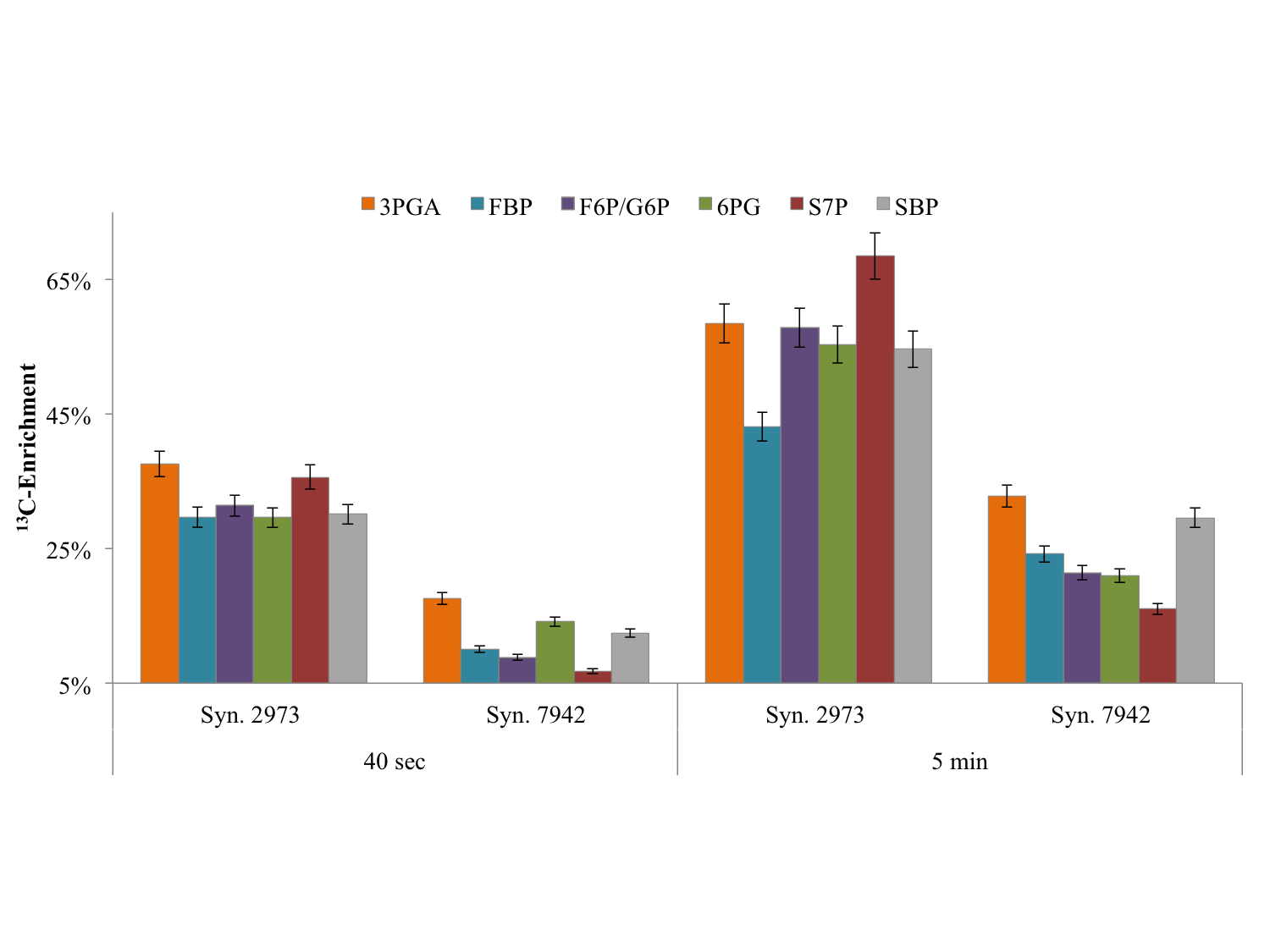
**

**Supporting Figure 7. Net fluxes fold changes of *Synechococcus* 2973 over *Synechococcus* 7942 net fluxes.** Fold change was calculated as the normalized net flux of 2973 over the normalized net flux of 7942 minus 1. Metabolic pathways and enzymes in the dark blue follow the y-axis scale on the left, while metabolic pathways and enzymes in light blue follow the y-axis scale on the right.

**
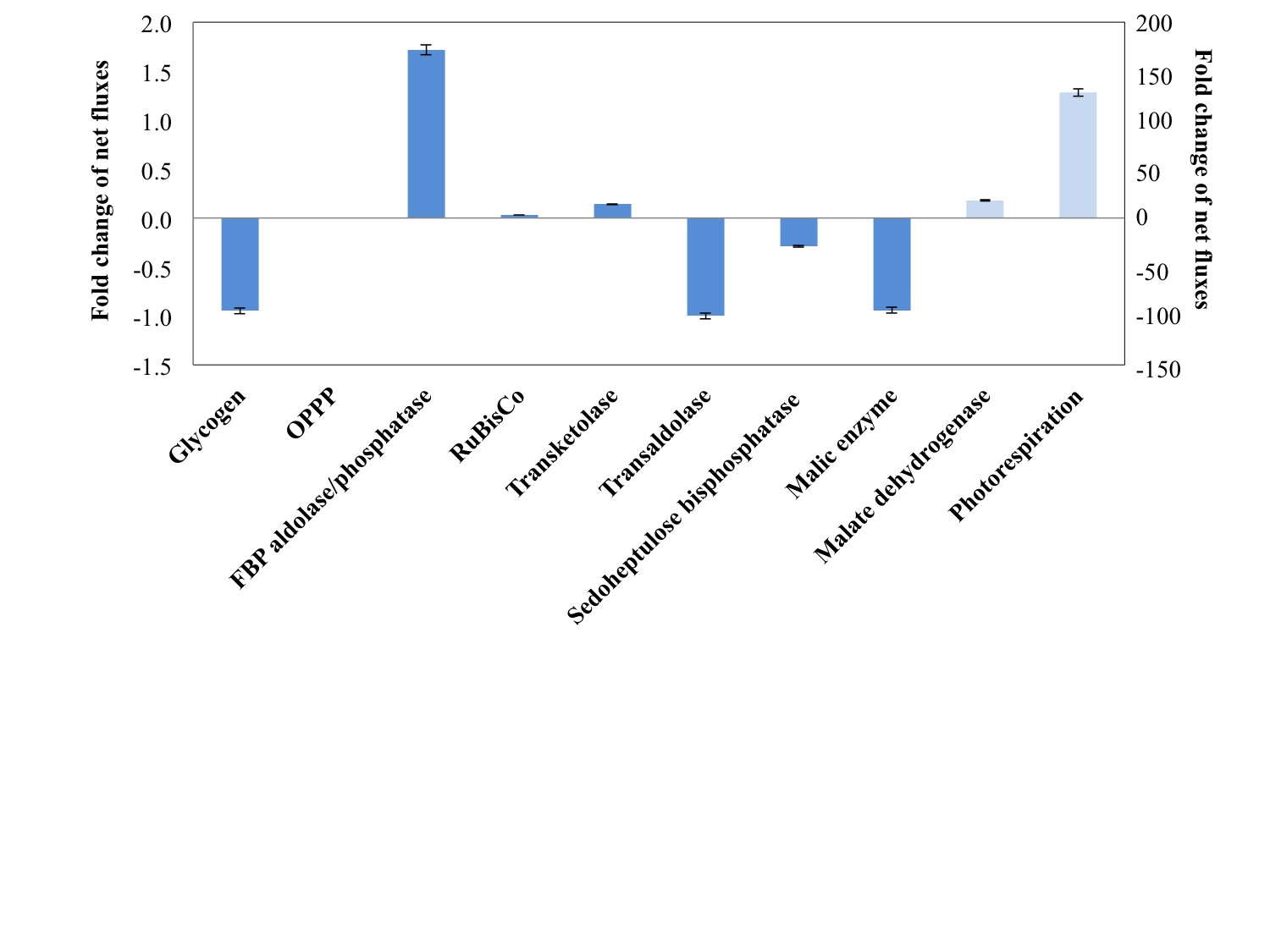
**

**Supporting Photo 1. Multi-cultivator system used to cultivate fast growing cyanobacteria, *Synechococcus* 2973.** The system is from Photon Systems Instruments (MC 1000) and uses cool white LEDs with adjustable irradiance.


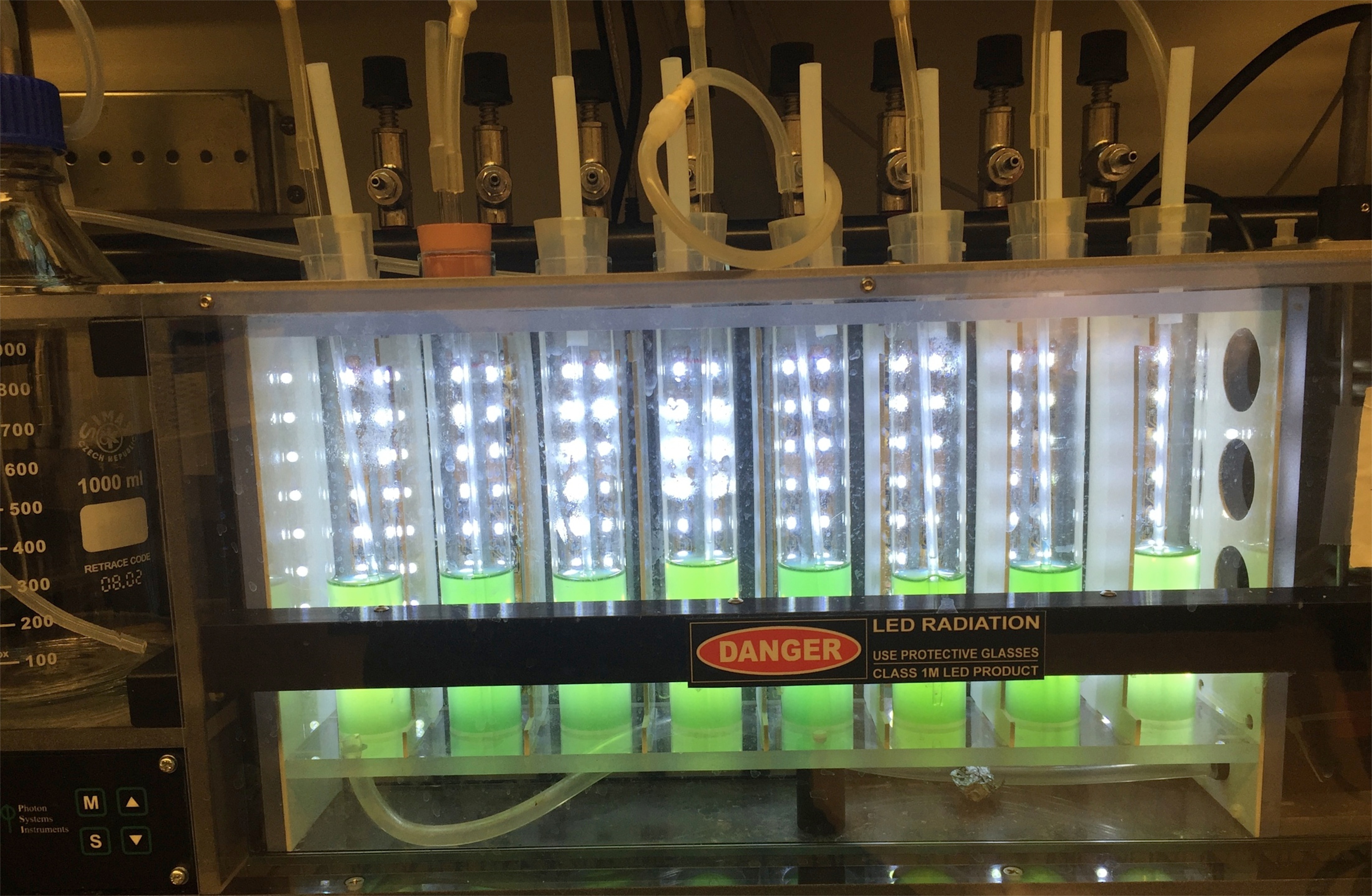


**References**

1. Seifter S, Dayton S, Novic B, Muntwyler E (1949) The estimation of glycogen with the anthrone reagent. *Arch Biochem* 25(1):191-200.

2. Porra RJ, Thompson WA, Kriedemann PE (1989) Determination of accurate extinction coefficients and simultaneous equations for assaying chlorophylls a and b extracted with four different solvents: verification of the concentration of chlorophyll standards by atomic absorption spectroscopy. *Biochim Biophys Acta BBA - Bioenerg* 975(3):384–394.

3. Schledz M, Seidler A, Beyer P, Neuhaus G (2001) A novel phytyltransferase from *Synechocystis* sp. PCC 6803 involved in tocopherol biosynthesis. *FEBS Lett* 499(1–2):15–20.

4. Shastri AA, Morgan JA (2005) Flux balance analysis of photoautotrophic metabolism. *Biotechnol Prog* 21(6):1617–1626.

5. Ma F, Jazmin LJ, Young JD, Allen DK (2014) Isotopically nonstationary ^13^C flux analysis of changes in *Arabidopsis thaliana* leaf metabolism due to high light acclimation. *Proc Natl Acad Sci* 111(47):16967–16972.

6. Quan J, Tian J (2011) Circular polymerase extension cloning for high-throughput cloning of complex and combinatorial DNA libraries. *Nat Protoc* 6(2):242–251.

7. Yu J, et al. (2015) Synechococcus elongatus UTEX 2973, a fast growing cyanobacterial chassis for biosynthesis using light and CO_2_. *Sci Rep* 5:8132.

8. Ungerer J, Pakrasi H (2-16) Cpf1 is a versatile tool for CRISPR genome editing across diverse species of cyanobacteria. *Sci Rep* 6:39681.

9. Nöh K, et al. (2007) Metabolic flux analysis at ultra short time scale: isotopically non-stationary ^13^C labeling experiments. *J Biotechnol* 129(2):249–267.

10. Xiong W, Brune D, Vermaas WFJ (2014) The γ-aminobutyric acid shunt contributes to closing the tricarboxylic acid cycle in *Synechocystis* sp. PCC 6803: The γ-aminobutyric acid shunt in *Synechocystis*. *Mol Microbiol* 93(4):786–796.

11. Zhang S, Bryant DA (2011) The Tricarboxylic Acid Cycle in Cyanobacteria. *Science* 334(6062):1551–1553.

12. Zhao J, Shimizu K (2003) Metabolic flux analysis of Escherichia coli K12 grown on ^13^C-labeled acetate and glucose using GC-MS and powerful flux calculation method. *J Biotechnol* 101(2):101–117.

13. Bennett BD, Yuan J, Kimball EH, Rabinowitz JD (2008) Absolute quantitation of intracellular metabolite concentrations by an isotope ratio-based approach. *Nat Protoc* 3(8):1299–1311.

14. Young JD, Shastri AA, Stephanopoulos G, Morgan JA (2011) Mapping photoautotrophic metabolism with isotopically nonstationary ^13^C flux analysis. *Metab Eng* 13(6):656–665.

15. He L, Wu SG, Wan N, Reding AC, Tang YJ (2015) Simulating cyanobacterial phenotypes by integrating flux balance analysis, kinetics, and a light distribution function. *Microb Cell Factories* 14:206.

16. Pramanik J, Keasling JD (1998) Effect of *Escherichia coli* biomass composition on central metabolic fluxes predicted by a stoichiometric model. *Biotechnol Bioeng* 60(2):230–238.

17. Yang C, Hua Q, Shimizu K (2000) Energetics and carbon metabolism during growth of microalgal cells under photoautotrophic, mixotrophic and cyclic light-autotrophic/dark-heterotrophic conditions. *Biochem Eng J* 6(2):87–102.

18. Xiong W, et al. (2015) The plasticity of cyanobacterial metabolism supports direct CO_2_ conversion to ethylene. *Nat Plants* 1(5):15053.
